# Supplementary material for: Conversational, Longitudinal, Ecological Assessment (CLEA): Exploring a new AI-driven method for qualitative data collection in a behavioural health context
Source: PLOS Digit Health. 2026 May 27;5(5):e0001216. doi: 10.1371/journal.pdig.0001216 (PMC13215495; doi:10.1371/journal.pdig.0001216)
Supplement: S2 File — (DOCX) [file pdig.0001216.s002.docx]

**Key Points – Semi-Structured Discussion Guide**

**Reasons for initial engagement & hopes for the programme**

- When did you first become aware of the Fit Robins programme?
- What made you decide to enrol? – followup with probes
- Did you have any concerns about joining the programme?
- Did you have any specific goals that you are hoping the programme will help you accomplish?

**Experience of digital diary method**

- What are your first thoughts when you get prompted by the chatbot for a Q&A session?
- What do you think about the questions it asks you?
- How do you feel after completing a Q&A session with the chatbot?
- Is there any difference in how you perceive the chatbot between the first time you used it, to now? Why?
- Do you think it could be improved in any way? Why would that improve it?
- Does the chatbot feel like a part of the Fit Robins programme to you?
- Do you think the chatbot helps you get more out of the programme in any way?
