# Supplementary material for: Conversational, Longitudinal, Ecological Assessment (CLEA): Exploring a new AI-driven method for qualitative data collection in a behavioural health context
Source: PLOS Digit Health. 2026 May 27;5(5):e0001216. doi: 10.1371/journal.pdig.0001216 (PMC13215495; doi:10.1371/journal.pdig.0001216)
Supplement: S3 File — (DOCX) [file pdig.0001216.s003.docx]

[
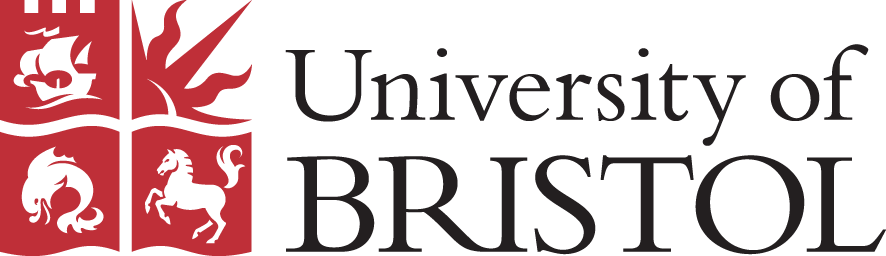
](https://www.google.com/url?sa=i&source=images&cd=&cad=rja&uact=8&ved=2ahUKEwjY26m9_7DbAhUJRhQKHb2UCc4QjRx6BAgBEAU&url=https://www.bristol.ac.uk/bilt/bilt-buzz/news/&psig=AOvVaw2hS4HciZQfZoOpUXEiOPT-&ust=1527892016821869)

**Data Protection Impact Assessment (DPIA) relating to:**

**[Title of Initiative/project/process/study/system etc]**

DPIAs should be sent to the Information Governance Team at [**data-protection@bristol.ac.uk**](mailto:data-protection@bristol.ac.uk)

DPIAs produced as part of the IT Services new service assessment procedure, or with an IT element, should also be send to the Information Security Team at [**cert@bristol.ac.uk**](mailto:cert@bristol.ac.uk)

**Document control**

| **Version** | **Date** | **Author** | **Summary of changes** | **Approver** | **Approval date** |
| --- | --- | --- | --- | --- | --- |
| 1 | 24/04/23 | Samuel Downes | Initial |  |  |
| 2 | 07/06/23 | Samuel Downes | Addition of technical amends including removing of Otter.AI |  |  |
| 3 | 27/06/23 | Samuel Downes | Amend to use Microsoft Stream for transcription services |  |  |
|  |  |  |  |  |  |
|  |  |  |  |  |  |

| **Part A: Summary of the Initiative** | | | | | | | | | |
| --- | --- | --- | --- | --- | --- | --- | --- | --- | --- |
| **Describe the scope of the Initiative** (to include its aims and objectives; business/research/other case; level of investment in terms of time, financial and other resources; duration and geographic reach; visibility within and outside the organisation) | | | | | | | | | |
| **Research aim:**  Understand the barriers and facilitators to regular physical activity for an underserved, lower-income population in South Bristol.  **Objectives:**   - Recruit 25 middle aged adult males from areas of deprivation in South Bristol in partnership with a local charity - Conduct mixed methods primary research, comprised of 1-on-1 interviews performed in person, as well as a digitally enabled ‘diary method’ that captures reflective insights into people’s relationship with physical activity, in their lived context. This ‘diary method’ will be supported by objective measurements of physical activity gathered remotely via the participant’s smartphone. - Gain insights into the unique needs of this target population, in a level of richness that has not been captured before.   The active study period will be a total of 10-14 days, depending on the scheduling availability of participants. Participants will be reimbursed with £100 Love2Shop voucher.  Insights gathered from this work will be used to inform the participatory development of a novel digital intervention that is co-designed around the distinct needs of this under-served population. The data behind these insights will be pseudonymised and stored in the encrypted SharePoint digital environment at the University of Bristol. | | | | | | | | | |
| **Status of the Initiative** (describe the current phase of development or implementation of the Initiative or, if the Initiative has already commenced, when it commenced and the extent to which the processing activities relating to the Initiative are still ongoing) | | | | | | | | | |
| As of May 2023, the study outline (the target population and the methods to engage them) have been co-designed with our charity partner, the Robins Foundation (charity arm of Bristol City Football Club). The digital system has also been prototyped and demonstrated to the charity team who are community ambassadors for the population we are looking to target. Iterations to the system based off feedback from the charity are currently being implemented. Development of training materials and guidance documents is currently underway that will be used for recruiting individuals from the community of interest. | | | | | | | | | |
| **Part B: Description of the processing** | | | | | | | | | |
| **Nature of the processing** | | | | | | | | | |
| **Method(s) of collection** (e.g. online or paper-based forms completed by data subjects or feeds from other systems) | | There are three separate core methods for data collection in this study.   1. In-person semi-structured interviews, where the interview is recorded through Microsoft Stream signed in through a university 365 account. 2. For the remote phase of the study, activity levels will be captured through Google Fit app on the participants’ smartphone, sent to our secure server hosted on Google Cloud services, processed, and subsequently stored in Microsoft Excel in the University’s secure digital environment (SharePoint run on Microsoft Azure). 3. Also in the remote phase, qualitative data from the ‘diary’ method will be captured via WhatsApp and similarly stored in excel, in combination with the physical activity data per participant.   Separate to these methods, we intend to use Microsoft Forms for our participant consent forms. These consent forms will subsequently be stored once completed in the University’s SharePoint, where they will be secured in a password protected folder accessible only to the core research team. | | | | | | | |
| **Source(s) of the personal data being processed** (if personal data originates from third party sources, describe them) | | ***Data Collection Method 1:***  For the remote phase, ***activity data*** is gathered through Google Fit app on Android/IOS. This ***activity data*** is sent through a custom web application developed for this study hosted on Google Cloud’s App Engine to Microsoft Power Automate in the University’s secure environment, to be stored in Microsoft Excel. Diary method ***qualitative data*** will be sent from WhatsApp on the participants’ smartphone, through Twilio, to Microsoft Power Automate and into excel in the University’s SharePoint. Data will also be held in a secure database in Google Cloud SQL, where a virtual machine will be running in a private IP environment accessible only by a service account from our web application running in Google App Engine. This database is necessary for the fast processing of conversation data our application requires – all data for each participant will be erased from the database once they have completed the remote phase. Past this, their data will persist only in the university’s secure 365 environment.  ***Data Collection Method 2:***  For the second method of data collection, the in-person interviews, the ***qualitative data*** originates from the data subjects directly, and is recorded via Microsoft Stream signed in through the researcher’s university 365 account. Microsoft Stream will generate the transcript which will subsequently downloaded and stored in the University’s secure SharePoint. The recording itself will be destroyed. | | | | | | | |
| **Matching or combination of datasets** (to what extent does the processing involve multiple datasets collected for separate purposes) | | ***Quantitative activity data*** (specifically, step counts and ‘activity minutes’) will be gathered for each individual throughout the study, in combination with the qualitative data captured from the diary method. These data will be taken together to inform the subsequent participant in-person interview. | | | | | | | |
| **Processing activities relating to the personal data** (how will personal data be processed after collection) | | After collection and storage as outlined above, ***qualitative data*** will be analysed as per the standard method of Thematic Analysis (for more information about this method, please [see this link](https://www.sciencedirect.com/topics/social-sciences/thematic-analysis)). The combined dataset of ***qualitative/quantitative data*** will be pseudonymised and held to inform the next stage after this study, co-design of a digital intervention to support this population with physical activity. It will be stored in the University’s secure Microsoft SharePoint environment. | | | | | | | |
| **Scope of data sharing with third parties** (you may want to refer to a data flow diagram or other materials explaining data flows) | | For the in-person interview, transcripts will be processed via Microsoft Stream from a research team member’s computer signed into a university 365 account.  The remote method of data collection is more involved and therefore a diagram has been produced to help describe the data flows.  As per diagram below, physical ***activity data*** is captured from a wrist-worn fitbit, onto the participant’s smartphone device and sent through Google Fit to our app running on Google Cloud App Engine. This data is then sent from our server to Excel via Microsoft Power Automate – in the University’s secure environment.  ***Activity data*** is pulled from excel, sent to our web application running in Google cloud, and used to inform the questions formed through OpenAI’s chat API. These questions are then sent through Twilio and WhatsApp to the participant’s phone.  Responses (the ***qualitative data***) are then handled by the same flow in reverse. WhatsApp sends the participant response to a question through Twilio to our web application hosted on Google Cloud App Engine, which pseudonymises the message and sends onto the OpenAI API to generate a response. Within the remote study period, conversation histories will be stored in a private PostgresQL database running in a private virtual machine instance in Google Cloud SQL – only our application has the necessary credentials to access these conversation histories, which it does through a secure SSH tunnel. The database has no public IP address.  The OpenAI model being used is ‘GPT3.5 Turbo’ and has been programmed to generate reflective questions that encourage users to share appropriate details about their relationship about physical activity, the answers users give are fed back to the model to form unique questions that are formed to learn more about an individual’s lived experience. The research team programmed the OpenAI model and extensively reviewed the model output which performs extremely well. The team are working on a safety mechanism whereby the participants can suspend the study with a chosen keyword if they ever feel an inappropriate question is generated. If this occurs, the research team will be alerted and will investigate the issue immediately  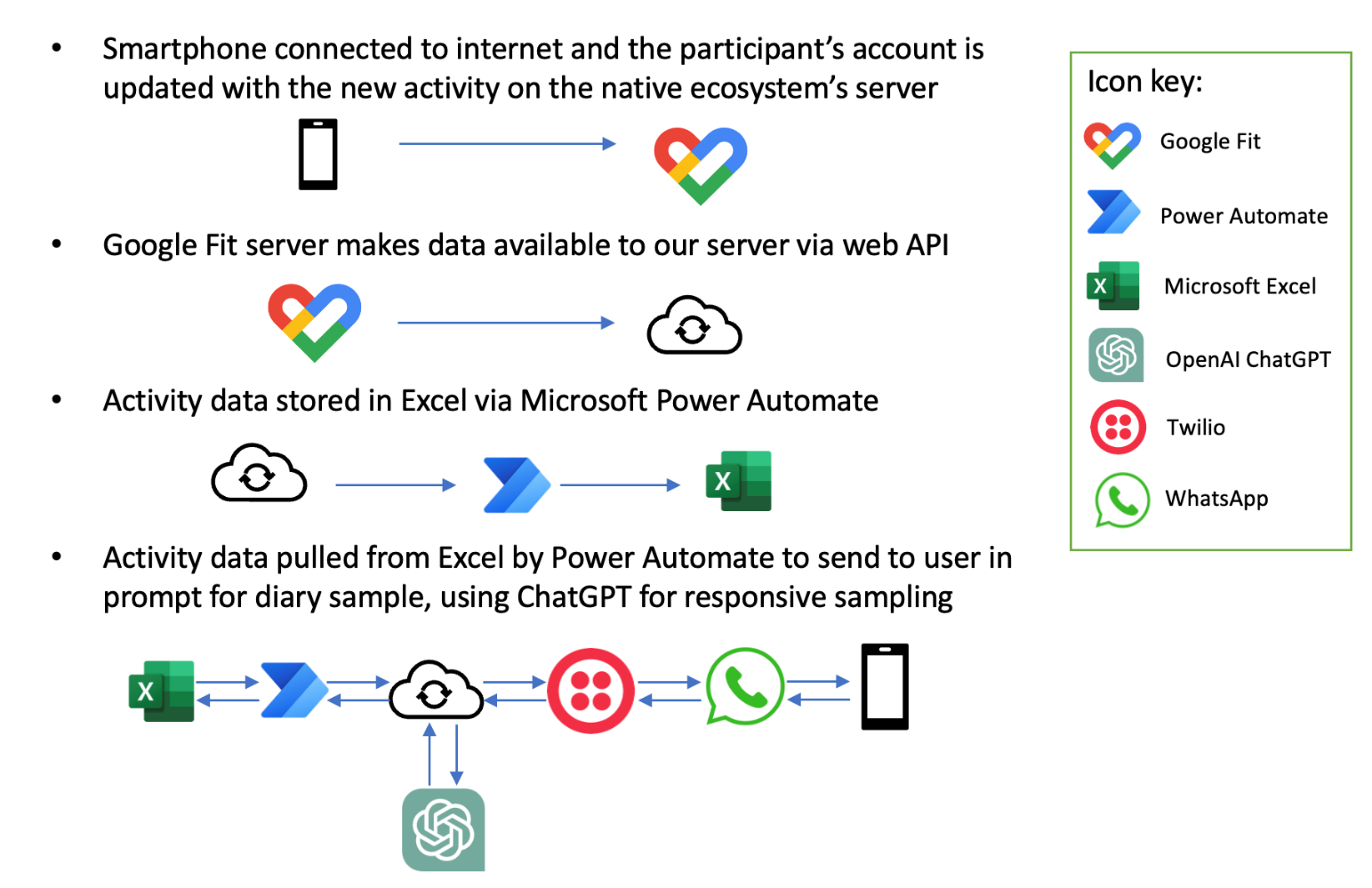 | | | | | | | |
| **Extent of automated decision-making** (describe extent to which decisions are made about data subjects without human intervention/review, e.g. through the use of automated algorithms) | | For the diary method, users schedule when they would like to be prompted for providing data samples. There will also be an algorithm that detects substantial periods of activity/inactivity to trigger sample collection asking participants to reflect on their current activity levels. The ‘diary method’ is performed via the specifically programmed OpenAI model to enable real-time reflective interviewing across participants simultaneously. The messages sent by the OpenAI model are strictly adherent to the carefully formed instructions agreed by the research team and charity partners to set clear parameters for the model’s generative ability specific to this study.  A contingency safety mechanism in the system will enable participants to immediately suspend any incoming messages from our application if they feel an inappropriate message has been sent to them. They can elect a keyword when they are onboarded to the study which they can simply type and send in the chat which will automatically pause the data-gathering and alert the research team to investigate the matter immediately. | | | | | | | |
| **Scope of the processing** | | | | | | | | | |
| **Categories of personal data** (identify each category of personal data processed, including any special category data and information relating to criminal convictions and offences) | | - Qualitative health-related data will be captured from the interview stage and via the what’s app responses. - Physical activity data will be collected from participants – specifically their step count and ‘active minutes’ recorded by Google Fit. - Qualitative data relative to their physical activity levels will be collected. - Personal, identifiable data gathered as part of the consent process and use of what’s app messages. - As the responses to questions sent via what’s app are free text, additional personal information may be processed if provided by the participants. | | | | | | | |
| **Categories of data subject** (e.g. staff, students, research participants, website visitors, device users, children, vulnerable adults) | | The data subjects will be all of the below:   - Consenting adults - Device users - Interview subjects | | | | | | | |
| **Format of the personal data** (e.g. paper records, electronic documents, spreadsheets, databases, system records or other files) | | Spreadsheets (Microsoft Excel), in University’s SharePoint.  Conversation histories will temporarily be held in a private databases (PostgresQL, running on Google Cloud SQL), being wiped completely once each participant completes their remote part of the study. | | | | | | | |
| **Storage location** (e.g. locked filing cabinets, document repositories, on-premise servers or storage devices, cloud-hosted services in UK, EU or international) | | Spreadsheets will be stored within the University’s secure digital environment and accessible only be credentialed staff from the research team. The participant’s identifiable information will be stored in this way. The Microsoft resources (Excel and Power Automate) run on Microsoft Azure cloud architecture hosted in UK.  The PostgresQL database for holding conversation histories will be located in a private IP environment in a virtual machine instance of Google Cloud SQL services, hosted in Western EU serverbase. | | | | | | | |
| **Duration and frequency of processing** (by reference to the relationship with the data subject or the nature of the Initiative) | | Data processing to be ongoing over 10-14 day study period, subsequently data will be pseudonymised, analysed and stored within the University indefinitely. | | | | | | | |
| **Volume of data subjects and records** (or an approximation where it is not possible to confirm precise numbers at present) | | 25 participants, each with daily records of activity levels and diary samples over the 10-14 day study period. | | | | | | | |
| **Context of the processing** | | | | | | | | | |
| **Relationship with data subjects** (describe the proximity between the University and the data subjects and how the relationship is established) | | Research team to be introduced to data subjects via local charity partners. Research team will meet with participants in their local community, alongside our charity partners.  Participants will be met by the research team a minimum of twice, with remote monitoring as per described methods ongoing between these meetings. | | | | | | | |
| **Data subjects’ expectations** (describe the extent to which the data subjects are aware of and expect their personal data to be used in connection with the proposed processing activities) | | Data subjects will be briefed by charity partners on the level of physical activity data being captured alongside the qualitative data captured via the diary method. They will also be provided with plain-english summaries of what is happening to their data both during the study and afterwards. Consent forms will be co-designed with charity partners so as to be clear and meaningful to participants. | | | | | | | |
| **Use of new technology or novel approach** (describe the extent to which the processing activities involve the use of any technology or other approaches that may be considered state of the art, novel or unexpected) | | OpenAI’s API chat model will be implemented at the core of our diary method, in a novel approach to qualitative research.  We have carefully considered the parameters set to moderate the model’s responses to users, setting strict rules at the system level of the model.  The programmed model has been diligently tested and provides high-quality and appropriate questions in response to user messages, that allow for extremely insightful samples to be captured, that otherwise could only be rivalled by traditional ethnography work requiring in-person observation.  A contingency for participants will be built-in to the system to allow them to suspend incoming messages immediately with a keyword phrase they choose.  The data sent to OpenAI will have the names of participants removed to pseudonymise the data. There may be some information provided in participant responses which could identify them even with their name removed but this is unlikely. | | | | | | | |
| **Relevant matters of public concern** (describe any matters of public concern relating to the scope of the processing or the use of any particular technology or approach, if applicable) | | There are wider societal and existential worries about the use of AI which may be of concern to some participants.  It is therefore critical for users to be well informed of why and how the AI is being implemented for this work, and to know that the purpose of this work is only to better understand the needs of people like them, so that in the future better interventions can be developed to support them in living more active, healthier lives. | | | | | | | |
| **Purposes of the processing** | | | | | | | | | |
| **Benefits to the data subject** (describe how the processing benefits the data subjects/individuals either directly or indirectly) | | Directly, capturing the personal information required as part of the consent process is necessary to enable the data subject to partake in the study, and to receive the monetary incentive of partaking.  Indirectly, in terms of the overall study aims, capturing the level of insight we hope to gain from this study will directly be used to inform the participatory development of a digital intervention to help other people from the specific population demographic to be more regularly physically active. | | | | | | | |
| **Benefits to the organisation** (describe how the processing benefits the organisation either directly or indirectly) | | Benefits to the university include reputational gains from utilising cutting edge technology to better understand the needs of underserved groups in terms of health and lifestyle, so that health inequalities can be effectively addressed.  For our charity partners, we are providing a mechanism for them to better understand the needs of the community they serve, so that they might develop ever-better programmes for community members moving forwards. | | | | | | | |
| **Benefits to third parties** (describe how the processing benefits any third parties either directly or indirectly) | | This research is intended to benefit this specific population demographic in engaging in regular physical activity. | | | | | | | |
| **Part C: Consultation process** | | | | | | | | | |
| **Input of internal stakeholders, experts and other professionals** (advice from parties including senior staff, specialists, IT experts, lawyers, security consultants, ethics advisers etc, where applicable) | | Project consulted by:   - University of Bristol Digital Health staff - Robins Foundation team (charity partner) - University of Bath Digital Health collaborator | | | | | | | |
| **Advice from Data Protection Officer** (where applicable, obtaining the advice of the DPO is a mandatory requirement – this may be set out in a separate appendix/document) | | Proactive support provided by University of Bristol DPO | | | | | | | |
| **Input from data subjects (or their representatives)** (where relevant describe the views sought, consultation methodology or justification for not seeking input) | | All of our study methods which involve interaction with data subjects are co-designed with the team from our charity partner. This is necessary because we are targeting a ‘hard to reach’ group, where relationships are critical to doing work. Our charity partners are carrying out the initial stages of the recruitment process (with input from the core UoB research team), and will subsequently introduce us to the data subjects. Our charity partners are also able to advise on study design given they come from areas of deprivation themselves and have a deep knowledge of the people who will participate in the study. | | | | | | | |
| **Part D: Assessment of necessity and proportionality** | | | | | | | | | |
| **Lawful basis for processing** (identify the most appropriate ground(s) for lawful processing, explaining the rationale - see Appendix 3 for permissible grounds. For legitimate interests a separate legitimate interest assessment is needed.) | | Lawful basis for this work is in accordance with:  6(1)(a) Consent: the individual has given clear consent for you to process their personal data for a specific purpose.  6(1)(e) Public task: the processing is necessary for you to perform a task in the public interest or for your official functions, and the task or function has a clear basis in law. The University’s public tasks revolve around teaching and research. All research can come under this lawful basis.  **Special category data**  9(2)(a) Explicit consent: the individual has given their explicit consent to the processing of their personal data for the specific purpose.  9(2)(d) Substantial public interest: the processing is necessary for reasons in the substantial public interest where it will safeguard the rights and interests of the individual.  9(2)(e) Medical purposes: the processing is necessary for the purposes of preventive or occupational medicine, or the provision of health care.  9(2)(c) Research purposes: the processing is necessary for purposes of scientific or historical research in the public interest. This lawful basis will apply to all research conducted by the University involving special category data. | | | | | | | |
| **Fairness and transparency** (describe the means by which data subjects will be informed about the intended processing, e.g. fair processing notices, technical notifications, consent forms, participant information sheets) | | Data subjects will be given an overview of the study by charity team members who are able to ‘speak their language’ – and thus convey meaningful content. We will also provide plain-english summaries, FAQ documents as well as formal consent forms and participant information sheets.  The consent form will clearly advise participants that the University as a whole has not approved the use of WhatsApp, Twilio or Chat GPT. The consent form will be explicitly clear that the use of these platforms is on a consent basis and that we have put measures in place to mitigate risks where we can such as psuedonymisation, deletion of personal data expeditently and as minimal transfer of personal data as possible. | | | | | | | |
| **Data minimisation** (describe the steps that will be taken to ensure that the amount of personal data is minimised and limited to what is strictly necessary both initially and on an ongoing basis) | | Out of all the physical activity data available via Google Fit, we are only requesting access to step counts and ‘activity minutes’. This is because step counts are a universal measure of general activity, and ‘activity minutes’ allow for collection of activity data that is not represented by step counts.  Personal data collected to understand participants baseline characteristics will strictly be relevant to potential barriers/facilitators of physical activity, and/or to establish demographic data (e.g., gender, age, income status, education level).  In order to contact participants we are only collecting phone numbers and email addresses. | | | | | | | |
| **Necessity of processing** (explain the extent to which the processing is necessary in relation to the purposes of the initiative) | | The processing of activity data is necessary to provide objective cues to users when they are asked about their physical activity levels.  The processing of responses that takes place in real time with the OpenAI API is to enable active listening and reflective questioning to understand the root barriers and facilitators of individuals.  Subsequent thematic analysis of qualitative data is necessary to separate ‘signal from noise’ in terms of learning generalisable findings within the target population. | | | | | | | |
| **Accuracy** (describe the steps taken to ensure data quality in terms of accuracy and freedom from bias, both initially and on an ongoing basis, e.g. verification techniques and how individuals can update their data) | | User’s physical activity data is updated at regular intervals to ensure prompts for samples via the diary method are rooted in accurate context of their recent physical activity.  Users can re-register at any point to update their contact details, e.g if they wanted to change phone number.  Participants will be made aware that they are able to erase all physical activity data stored through use of Google Fit app. | | | | | | | |
| **Storage limitation** (describe the steps taken to ensure that personal data are not retained longer than necessary in connection with the intended purposes of the processing) | | The credentials to access participant activity data will be wiped at the end of the study. Individuals may choose to keep Google Fit but Bristol University will no longer have our application connected to their account after study completion. A permanent erasure request will be made to Google Cloud SQL services at the end of the study for any residual user account information. The cloud project itself will be deleted along with registered user details. T  The collected qualitative and quantitative data will be pseudonymised and stored securely within the encrypted digital SharePoint environment provided to the university by Microsoft Azure services. Identifiable data taken as part of the consent and formal recruitment process via Redcap will not be stored permanently, being deleted after a period of 6 months following the study’s conclusion. This is so that we have relevant contact details if needed for immediate safety or follow up, or if a request is made to remove an individual’s contribution to the data pool.  Requests will be made to Twilio at the end of the study to wipe residual message data that it has processed as part of the study held within the researchers accounts.  The Postgres SQL database (run on Google Cloud services) used to manage message data will be wiped at the end of the study. | | | | | | | |
| **Security, integrity and confidentiality** (describe the steps taken to ensure the security of the personal data, including protection against personal data breaches) | | Participant personal identifiable information will be stored in an excel spreadsheet in the University of Bristol’s Microsoft SharePoint digital environment.  During the study, data will be collected and temporarily stored in a Postgres SQL database hosted in a private Google Cloud SQL virtual machine instance. All of this data will be wiped at the end of the 10-14 day study period. The database can only be accessed by our app running in Google Cloud App Engine, through a secure SSH tunnel to the private IP. | | | | | | | |
| **Data subject rights** (describe the steps taken to ensure that data subjects are able to exercise their rights fully and effectively. Individuals have the right to be informed, and rights of access, rectification, erasure, objection and to stop automated decision making) | | We will co-design participant information sheets and consent sheets with our charity partners who can advise on the correct language needed to be confident the target participants will have full opportunity to understand their data rights as a participant of the research. | | | | | | | |
| **Third party processors** (where relevant, describe the steps taken to ensure the reliability of third parties processing the data on the University’s behalf, and their compliance with data protection law) | | Participant data is held on Google Cloud services, and Microsoft Azure (on the University’s secured digital environment). Within Google Cloud, our application is run on Google Cloud App Engine and conversation history is stored (for the 2 week remote phase only) in a PostgresQL database run in a virtual machine instance in Google Cloud SQL. This SQL database does not have an openly accessible IP address. It has a private IP address and can only be accessed by a credentialed request made by the service account connected to our web application. When our web application retrieves conversation histories, it creates a secure private SSH tunnel using our credentialed service account to connect to our database. We will erase all data from the SQL database at the end of each participant’s remote phase period (max 2 weeks). Thereafter, the data we capture from participants will only be stored in the university’s secure environment.  Both Google and Microsoft state compliance with GDPR and recognised international standards for data protection and privacy. In particular, with ISO 27017 (Cloud security) and ISO 27018 (Cloud privacy) which are of key relevance.  OpenAI does not store the messages sent to it via API. The service they provide is run through their own cloud on Microsoft Azure cloud computing services.  Twilio will temporarily store message data and participant phone numbers in their Amazon Web Services (AWS) cloud infrastructure. The AWS cloud storage used is encrypted with high security standard – 256-bit Advanced Encryption. Data will be erased from Twilio and AWS at the end of the study period.  WhatsApp stores message data locally via encrypted backups on the user’s device. WhatsApp is GDPR compliant. User’s phone number, device ID and IP address may be shared with WhatsApp’s parent company, Meta. Participants will be made aware during the consent process that the university cannot guarantee the security or privacy of data handled by these third parties.  The University as a whole has not approved the use or these third parties or entered into contracts/data processing contracts with them. Participants will be aware of this through the consent form and this will be explained to them as well at point of gaining consent. | | | | | | | |
| **International transfers** (identify any international transfers of personal data, whether or not to a third party processor, and the safeguards implemented in relation to such transfers) | | Twilio, who are managing the WhatsApp messaging component, use servers in the US which will be directly relaying and forwarding messages to participants.  Twilio is GDPR compliant and has good history of use cases within research and proactive support for researchers. | | | | | | | |
| **Part E: Identification and assessment of risks (see Appendix 1 and Appendix 2 for example risks and assessment process)** | | | | | | | | | |
| **Ref No** | **Source of risk and potential impact on data subjects** (including associated compliance and organisations risks) | **Likelihood of harm** (see Appendix 2) | | | **Impact of harm** (see Appendix 2) | | | **Overall risk** (low, medium, high) | |
|  | Data leakage; private data from Data Subjects leaking from utilised databases or during data processing. | 1 | | | 4 | | | Medium - We have taken steps to maximise security of data we need to hold temporarily outside the university’s environment in the form of a separate, private SQL database that can only be accessed by the credentialed service account connected to our web application through a private SSH tunnel. Futhermore, data held long-term will be fully anonymised. The third parties involved provide information on how they meet GDPR compliance - we are gathering and processing data within digital environments that have demonstrated high security (evidenced by compliance of key service providers Google Cloud and Microsoft Azure with recognised standards). However with no University approved data processing contract we cannot provide any further mitigation or protection for the personal data processed by these third parties. Any terms and conditions accepted on a free trial basis have not been agreed by the University as a whole. | |
|  | Data Subjects agree to study conditions without full understanding of what happens to their data | 4 | | | 3 | | | Medium – see below | |
|  | Participants may opt to send voice messages during the remote part of the study, there is the risk that potentially sensitive information may be overheard and used against them by third parties. | 3 | | | 3 | | | Medium – see below | |
|  | Participants may share information beyond the scope of the study, that may still be highly sensitive. | 4 | | | 3 | | | Medium – see below | |
|  | Private data being shared with unsolicited third party apps or services. | 2 | | | 3 | | | Low – utilised apps/services have clear documentation of additional third parties who have a role in supporting their operations (e.g. OpenAI using Azure cloud services). It is unlikely the services used would put their reputations at stake and risk regulatory punishments by failing to document the additional third parties involved in running their services. | |
|  | Participant devices could be infected with spyware/malware which make the devices unsecure for data collection | 1 | | | 5 | | | Medium – see below | |
| **Part F: Identification of controls and measures to eliminate or mitigate risk (of medium or high risks items in Part E)** | | | | | | | | | |
| **Ref No** | **Controls or measures to eliminate or mitigate risk** (changes to design or additional safeguards and measures | **Effect on risk** (extent to which risk is eliminated or mitigated by the controls or measures) | | | | **Residual risk** (any risk remaining after controls or measures have been implemented) | | | |
|  | Mitigation of Part E ref.2:  Participants will be recruited by charity partners who they will have existing rapport with, and can communicate study details clearly to them. Plain-english summaries of study process will be provided to participants. Consent documents will be co-designed with charity partners to ensure language is appropriate/meaningful to participants. | The planned mitigation should be sufficient in ensuring that participants have a full opportunity to understand the full process and connotations of what they will be consenting to. | | | | There is the residual plausible scenario that potential participants may not pay close attention to the carefully tailored information made available to them, and still consent to participate. | | | |
|  | Mitigation of Part E ref.3:  Participants can opt to send typed messages instead of voice messages in this scenario. Participants will be made aware of their options and reminded to not share sensitive information out-loud if they do not feel they are in a private surrounding. | Participants will be informed of risk and provided with an alternative method to cut the risk substantially. | | | | There is still the risk that a typed message might be seen by an unsolicited third party but this risk is substantially smaller than voice. | | | |
|  | Mitigation of Part E ref.4:  The goal of this research is to better understand the day-to-day lives of our target population as it relates to physical activity. We will be asking exploratory questions that participants answer openly. Therefore it is very likely we will receive sensitive personal details that may not be related to physical activity. To minimise this, we have programmed the questions generated to explicitly focus on physical activity. | As questions will only probe participants to reveal information relevant to physical activity, participants are discouraged from sharing ‘extra’ information. | | | | Ultimately the nature of open-ended questions means it is possible for participants to provide a broad range of information – but open-ended questions are strictly favoured for this type of research. | | | |
|  | Mitigation of Part E ref.6:  Research team can check whether device has been ‘rooted’ or ‘jailbroken’ to determine whether there is a chance of spyware/malware on the device.  If device is jailbroken the device will not be usable in the study.  If devices have not been rooted or jailbroken, it is reasonable to assume they are not infected. However, further checks can be done such as ensuring firmware is up-to-date. | While the risk is not eliminated, if we are able to ensure all devices taking part in the study have not been tampered with (rooted/jailbroken), the chance of a partaking device running with spyware or malware is drastically lessened. | | | | There is a residual risk that participant’s devices have some sort of malware or spyware, ultimately it is up to the participants to ensure their personal devices are free from malicious software. | | | |
|  |  |  | | | |  | | | |
| **Part G: Implementation and integration of controls and measures** | | | | | | | | | |
| **Action** | | **Approved by** | **Person(s) responsible** | | | | **Target completion date** | | **Completed** |
| Review of information given when obtaining consent as the process continues to identify any common areas of confusion ensuring a transparent approach | | Michelle Radcliffe |  | | | |  | |  |
|  | |  |  | | | |  | |  |
|  | |  |  | | | |  | |  |
|  | |  |  | | | |  | |  |
|  | |  |  | | | |  | |  |
| **Part H:** **Outcomes and sign-off** | | | | | | | | | |
| **Residual risks that cannot be eliminated or mitigated (if any)** | | This study has been designed to access a hard-to-reach population. Due to these third-party providers have been selected as they will best meet the needs of this project, however these are not University approved providers. The participant consent form will clearly advise participants that the University (as a whole) has not approved the use of What's App, Twilio & Chat GPT and that they consent to using these providers. The University has worked to mitigate these risks where possible through pseudonymisation of data to Chat GPT, asking participants to delete What’s app chat history daily and by reviewing the privacy information available from all providers.  Extensive review has been carried out by the research team of the model output for the OpenAI generative questions with a safety mechanism built in and human oversight.  The risks of not having data processing contracts in place with these third-party providers have been accepted by the team as these are essential to this study and necessary to engage with this specific population. This is a balance between the privacy risks and the benefits to the wider population of this research being progressed, this has been carefully reviewed and mitigation to these risks is in place.  Michelle Radcliffe (Senior Information Compliance Officer) | | | | | | | |
| **Consultation with ICO** (where there are any residual high risks that cannot be eliminated or mitigated) | | **Date submitted** | |  | | | | | |
|  |  | **Submitted by** | |  | | | | | |
|  |  | **Outcome** | |  | | | | | |
| **Consideration of Data Protection Officer’s advice** (confirm whether advice accepted and implemented or rejected, and if rejected the reasons why) | |  | | | | | | | |
| **Sign-off** | | **Name and role** | | (Senior Information Compliance Officer) | | | | | |
|  |  | **Date** | | 28/06/2023 | | | | | |
| **Frequency of review (usually at least annually)** | | I would recommend a review during the process of collecting consent as per part G but following this there will be no need to review unless the study is expanded, or any processing of personal data is changed. | | | | | | | |
| **Next review date** | |  | | | | | | | |

**Appendix 1** **– Example types of risk associated with the processing**

Risks to data subjects

- Risk of processing being unlawful and/or regarded as unfair due to more personal data being collected than is necessary for the intended purposes of the processing
- Risk of personal data being inaccurate due to collection or processing methods or the nature of the personal data being processed
- Risk of personal data being retained longer than necessary or not properly managed so that duplicate records are created
- Risk of personal data being inadvertently manipulated due to human error or otherwise
- Risk of personal data being disclosed or accessed inappropriately due to inadequate access and disclosure controls
- Collection of personal data may be regarded as unnecessary and/or overly intrusive having regard to the objectives of the Initiative
- Risk of processing being unlawful and/or regarded as unfair due to scope and purposes of processing being extended inadvertently
- Use of new technologies, approaches or methods may constitute an unjustified intrusion on the data subjects’ right to privacy
- Risk of processing being regarded as unfair due to complexity of processing activities/involvement of algorithmic analysis
- Risk of processing being regarded as unfair due to the combination of matching of multiple datasets
- Identifiers may be collected and linked which prevent data subjects from accessing or using a service anonymously
- Collection of personal data and linking identifiers may result in anonymisation being compromised
- Vulnerable data subjects may be particularly concerned about risks of identification or disclosure of personal data
- Processing of personal data may produce legal effects or similarly significantly affect the rights and interests of the data subject
- Processing of personal data may result in inappropriate inferences being made or discrimination being suffered by the data subject
- Disclosure of personal data may result in discrimination, victimisation and/or harassment

Compliance risks

- Non-compliance with data protection laws, including the GDPR, Data Protection Act 2018, Privacy and Electronic Communications Regulations and other secondary legislation
- Non-compliance with common law duty of confidentiality
- Non-compliance with the Equality Act 2010 and other equality and human rights legislation
- Non-compliance with sector-specific legislation or standards

Associated organisational risks

- Risk of regulatory sanctions and fines
- Risk of reputational damage
- Risk of considerable financial expenditure to mitigate any risk that has materialised
- Risk of erosion of trust and confidence in processing activities resulting in loss of business
- Risk of investment returns being reduced or eliminated
- Risk of inaccurate, incomplete or outdated personal data having reduced value
- Risk of research or statistical objectives being compromised, skewed or false
- Risk of claims from individuals for compensation

**Appendix 2** **– Risk assessment methodology**

**Evaluation of likelihood of harm**

| **Likelihood score** | **1** | **2** | **3** | **4** | **5** |
| --- | --- | --- | --- | --- | --- |
| **Description** | **Rare** | **Unlikely** | **Possible** | **Likely** | **Almost certain** |
| **Frequency** | Will probably never happen | Not anticipated to happen, but possible | Might happen or recur occasionally | Will probably happen or recur, but not persistently | Almost certain to happen or recur, possibly frequently |

**Evaluation of impact of harm**

#

| **Likelihood score** | **1** | **2** | **3** | **4** | **5** |
| --- | --- | --- | --- | --- | --- |
| **Description** | **Very Low** | **Low** | **Medium** | **High** | **Very High** |
| **Impact** | Unlikely to have any impact | May have an impact | Likely to have an impact | Highly probably it will have a significant impact | Will have a major impact |

**Overall evaluation of risk**

| **Impact** | **Very High (5)** |  |  |  |  |  |
| --- | --- | --- | --- | --- | --- | --- |
|  | **High (4)** |  |  |  |  |  |
|  | **Medium (3)** |  |  |  |  |  |
|  | **Low (2)** |  |  |  |  |  |
|  | **Very Low (1)** |  |  |  |  |  |
|  | | **Rare (1)** | **Unlikely (2)** | **Possible (3)** | **Likely (4)** | **Almost certain (5)** |
|  |  | **Likelihood** | | | | |

**Appendix 3 – Lawful basis for processing personal data**

**Personal data**

The lawful bases for processing are set out in Article 6 of the GDPR. At least one of these must apply whenever you process personal data:

(a) Consent: the individual has given clear consent for you to process their personal data for a specific purpose.

(b) Contract: the processing is necessary for a contract you have with the individual, or because they have asked you to take specific steps before entering into a contract.

(c) Legal obligation: the processing is necessary for you to comply with the law (not including contractual obligations).

(d) Vital interests: the processing is necessary to protect someone’s life.

(e) Public task: the processing is necessary for you to perform a task in the public interest or for your official functions, and the task or function has a clear basis in law. The University’s public tasks revolve around teaching and research. All research can come under this lawful basis.

(f) Legitimate interests: the processing is necessary for your legitimate interests or the legitimate interests of a third party unless there is a good reason to protect the individual’s personal data which overrides those legitimate interests. This cannot apply if the University is processing data to perform its public tasks. A legitimate interests assessment may be required.

**Special category data**

If you are processing special category data (information about an individual’s race, ethnic origin, political opinion, physical or mental health, religion, trade union membership, genetics, biometrics, sexuality or sex life) then you also need a further lawful basis set out in Article 9 of GDPR. At least one must apply whenever you process special category data. The main Article 9 lawful bases are outlined here, though others also exist. Please seek further advice from the Data Protection Officer if required:

(a) Explicit consent: the individual has given their explicit consent to the processing of their personal data for the specific purpose.

(b) Employment law: the processing is necessary for pursing obligations set out in employment law.

(c) Vital interests: the processing is necessary to protect someone’s life where they are incapable of giving consent.

(d) Substantial public interest: the processing is necessary for reasons in the substantial public interest where it will safeguard the rights and interests of the individual.

(e) Medical purposes: the processing is necessary for the purposes of preventive or occupational medicine, or the provision of health care.

(c) Research purposes: the processing is necessary for purposes of scientific or historical research in the public interest. This lawful basis will apply to all research conducted by the University involving special category data.
