# Supplementary material for: Conversational, Longitudinal, Ecological Assessment (CLEA): Exploring a new AI-driven method for qualitative data collection in a behavioural health context
Source: PLOS Digit Health. 2026 May 27;5(5):e0001216. doi: 10.1371/journal.pdig.0001216 (PMC13215495; doi:10.1371/journal.pdig.0001216)
Supplement: Attachment — Submitted filename: Reviewer_Response_CLEA_SD3.pdf [file pdig.0001216.s006.pdf]

23rd April 2026

Response to Editorial Team: Conversational, Longitudinal, Ecological Assessment (CLEA): Exploring a new AI-driven method for qualitative data collection in a behavioural health context

Dear Editorial Team,

We thank you for your time and thoughtful feedback in reviewing our manuscript. We are grateful for the constructive comments which have helped us improve the quality of communication within the manuscript, and to fully surface the substance of our work. On a small number of occasions, reviewers' comments do not clearly align; when this occurs, we have sought to find an appropriate balance in the respective changes implemented in the manuscript. We have made direct reference to these instances in this document and justified our positions.

The following provides a point-by-point response to each comment. Reviewer's comments that were attached as separate documents have been incorporated into this response for ease of reference. All changes have been incorporated into the revised manuscript and are highlighted in tracked changes for your convenience. We hope that reviewers' feel we have suitably addressed their comments, and look forward to hearing back from the Editor.

Best wishes,

Samuel Downes,

On behalf of the authors

23rd April 2026

REVIEWER 1 (R1)

Thank you for the opportunity to review this thoughtful and timely manuscript. The study addresses a highly relevant methodological question in digital health research: how conversational AI can be deployed for longitudinal qualitative data collection in real-world behavioral health settings. The manuscript is well structured, grounded in relevant literature, and reflexive regarding technological design choices. That said, several areas require clarification and strengthening before publication. My review is uploaded as an attachment

This manuscript addresses an important and timely methodological question within digital health, qualitative methods, and human–AI interaction: how conversational AI may function as a tool for longitudinal qualitative data collection in real-world health contexts. The authors propose CLEA as a novel method and present an exploratory deployment within a community-based weight management program. The work is thoughtful, well written, and grounded in relevant literature across EMA, diary methods, and conversational agents. At the same time, because the manuscript makes methodological claims rather than reporting a conventional intervention outcome, the standards for conceptual clarity, transparency, and evidentiary alignment are especially high. Several sections would benefit from sharper framing, clearer differentiation between feasibility, acceptability, and utility, and more cautious interpretation of findings given the small, homogeneous sample and the entanglement between data collection and intervention effects. My comments below are intended to strengthen the manuscript's clarity, rigor, and contribution, not to undermine its value.

Abstract

1. The abstract is generally clear and informative, but it currently overstates the strength and generalizability of the findings given the exploratory nature of the study.

The abstract states: "Together, these findings demonstrate initial feasibility and acceptability of CLEA for longitudinal qualitative data collection in an underserved population." While the population is described as underserved, the sample is small (N=9) and predominantly White British (n=7). The authors should either: Clarify what is meant by "underserved" in this specific context, or Use more cautious language (e.g., "participants enrolled in a community-based program in an area of deprivation").

>>Author response

We have amended the abstract from

> "Together, these findings demonstrate initial feasibility and acceptability of CLEA for longitudinal qualitative data collection in an underserved population…

To

> "Together, these findings suggest initial feasibility and acceptability of this CLEA implementation within a community-based programme in an area of deprivation."

We have also amended the paper to make this clarification throughout the methods, results and discussion.

---

2. The abstract also claims CLEA can "elicit meaningful, contextually grounded insights consistently over time."
Given that reflective depth scores averaged 0.46/1, this claim would benefit from qualification, for example, by distinguishing session-level aggregation from individual response depth, which is discussed later in the paper but not reflected in the abstract. Tighten claims to more closely reflect the exploratory design and analytic results.

>>Author response

We have amended text from

> "These findings… illustrate its capacity to elicit meaningful, contextually grounded insights consistently over time."

To

> "Further, while the responses in single messages were often brief, useful and relevant, meaningful insights appeared to develop over the course of conversational sessions."

Introduction

3. The introduction is strong in its methodological grounding and provides a solid overview of existing longitudinal qualitative methods. However, several improvements would strengthen its contribution.

Positioning of CLEA as a "new method" The manuscript introduces CLEA as a "novel" method, but novelty is sometimes implied rather than explicitly demonstrated. The authors should more clearly articulate:

What CLEA enables that existing EMA + diary hybrids do not, and Whether the novelty lies primarily in the technology (LLM-based CA), the interactional structure, or the methodological framing.

>>Author response

We value Reviewer 1's feedback on the introduction and have addressed their comments, while acknowledging Reviewer 3's assessment that the introduction was solid and comprehensive.

We have now made this more explicit, adding the following:

> "In summary, qualitative interviews enable adaptive, in-depth data collection but are resource-intensive, often infrequent and removed from the moment of experience.

> Intensive longitudinal research (ILR) methods allow frequent, in-context data capture closer to events, but are static, varying from constrained (EMA) to unstructured (diaries). Integrating conversational AI into an IRL approach offers the potential to integrate some of the strengths of interviews, through enabling flexible, adaptive interactions with enough structure to stay on topic, while enabling repeated, in-the-moment data collection."

We have also amended the following:

> "We position CLEA as a high-level IRL method that, through specific design choices, can be adapted to conduct longitudinal research with varying qualities of EMA or diary method depending on requirements of the respective study context."

To

> "We position CLEA as a technological adaptation to IRL methods that, through specific design choices, can be adapted to conduct longitudinal research with varying qualities of EMA and/or diary method depending on the requirements of the respective study context."

---

4. The introduction states: "There has been limited investigation into the deployment of CAs for longitudinal, in-the-wild qualitative data collection…" This is accurate, but the authors could strengthen the argument by:

- **Explicitly stating what remains unknown (e.g., engagement decay, data quality trade-offs, reactivity), and**
- **Mapping each unknown directly to the stated aims (feasibility, acceptability, utility).**

>>Author response

We have added:

> "Specifically, little is known about how the acceptability, feasibility, or utility of AI-mediated qualitative agents might vary over time or be influenced by design choices."

And

> "The previously detailed works demonstrate that the capability of CAs to engage in interactive and dynamic conversation led to more informative, detailed responses from participants relative to static, traditional survey approaches. Whether this utility extends to longitudinal methods in health contexts is yet to be established."

These gaps map onto the aims already outlined in the introduction and methods (exploring (a) feasibility, (b) acceptability (c) utility).

---

5. The manuscript references a “constructivist consideration of knowledge production” later, but this stance is not clearly introduced here. Because epistemology directly shapes the interpretation of AI-mediated data collection, it would be helpful to:

- **Briefly signal the authors’ epistemological positioning earlier, and**
- **Explain how this stance justifies CLEA’s conversational and potentially interventional qualities.**

>>Author response

To address the point of epistemological positioning, we have added the following to the introduction to signal this early in the paper.

> “We adopt a pragmatic approach for guiding methodological decisions that embraces the co-constructive nature of qualitative data, and the interpretative manner of qualitative analysis.”

We also now include a positionality statement in the methods section (discussed more fully in response to reviewer 3, comment 3).

In relation to the point about addressing potential interventional qualities in the introduction, we discuss this more fully in our response to comment 9; however,, we did not foresee, or anticipate interventional effects, therefore we feel this emergent finding is best placed in the results and discussion.

Methods

Overall, the methods section is detailed and transparent, which is a major strength. However, several areas require clarification or strengthening.

6. The manuscript describes the study as “mixed-methods,” but the integration between quantitative engagement metrics and qualitative interview data is not fully articulated. Clarify whether this is:

- **A convergent mixed-methods design,**
- **A primarily qualitative study with descriptive quantitative support, or**
- **An exploratory methodological evaluation.**

>>Author response

This study has multiple aims, and as pragmatists, we chose the research methods that best address these [1]. Our mixed-methods approach used qualitative and quantitative approaches in parallel, independent of each other during analysis, with both sources of data considered during interpretation in the discussion [2]. We have clarified our mixed-methods approach, but adding the following in the study design section:

> "This is a mixed-methods study, with quantitative and qualitative components undertaken in parallel, with different methods chosen to address the different research aims; quantitative analysis of the chat logs to explore longitudinal engagement (aim A), content analysis of chat logs to explore clarity, relevance, depth and utility (aims A and C), and analysis of qualitative interviews to explore users' experiences and perceptions around acceptability, usability and utility (aims B and C)."

---

7. Eligibility criteria include "stable housing" and English-speaking participants. These criteria may systematically exclude individuals most affected by digital exclusion or instability. Acknowledge how these criteria shape the conclusions about accessibility and acceptability.

>>Author response

We have updated the limitations section of the discussion, which now reads as follows:

> "This is a mixed-methods study, with quantitative and qualitative components undertaken in parallel. Different methods were chosen to address the different research aims: quantitative analysis of the chat logs to explore longitudinal engagement (aim A); content analysis of chat logs to explore clarity, relevance, quality and utility (aims A and C); and analysis of qualitative interviews to explore users' experiences and perceptions around acceptability, usability and utility (aims B and C)."

---

8. Participants joined at different weeks (weeks 3–5). While acknowledged later as a limitation, this staggered entry has implications for longitudinal comparability that could be more clearly addressed upfront.

>>Author response

We have expanded on our description of sampling and recruitment in the methods section, which now states:

> "Sampling was opportunistic via the programme. Most participants (n=5) received study information in week 1, were invited to provide informed consent from week 2, and were onboarded from week 3. Additional participants were onboarded in weeks 4 (n=3) and 5 (n=1). This staggered approach was chosen to increase access to participation for those who missed early programme sessions or required more time to consider participation. However, it resulted in a shorter period of technology use for some participants."

---

9. CLEA System and AI Model Choice This is one of the strongest sections of the paper, but also one of the most methodologically consequential. The authors state: “The GPT4-Turbo model tends towards a positive, affiliative tone… This tendency… was desirable…” This choice is defensible, but it is also interventional, as later acknowledged. The methods section would benefit from:

- **A clearer justification of why an affiliative model was preferable to a more neutral one for a data collection study, and**
- **An explicit acknowledgement that this choice may shape participant affect and responses from the outset.**

>>Author response

The primary justifications for model choice were performance and safety. The comment about desirability was related to safety rather than affiliative tone, we stated: “GPT4-Turbo model tends towards a positive, affiliative tone and leans towards ‘safe’ interactional practices - avoiding overly sensitive or emotional topics [29, 30]. This tendency towards ‘safe’ interactions was desirable in this instance, considering typical in-person ‘distress protocols’ such as reading non-verbal cues are not applicable here [31].” We subsequently noted in piloting that the charity partners felt the affiliative tone was likely to be suited to users, and we also note in the methods that this was a known characteristic of the model.

To clarify that the primary justifications were around performance and safety, we have added the following details to the methods to justify the choice of model:

> “Technology choices were guided by principles of performance and safety, with consultation from charity partners, advocating for user needs. Considering performance, model selection was constrained by the technical landscape at the time of development (late 2023). Within this context, GPT-4 Turbo demonstrated reliability in consistently attending to information across longer prompts, follow system instructions reliably, maintain conversational coherence, and remain aligned to the intended research scope . GPT-4-Turbo was prioritised for prototype testing with charity partners in these respects, considering its preferable performance in terms of fidelity, which has implications for safety – for example, fidelity to adhering to guardrails like do not give medical advice. A prototype was set up in a test environment and connected to WhatsApp installed on university-owned mobile phones. Charity staff attended an in-person testing session, during which they interacted with the prototype live and provided feedback on its perceived suitability. In particular, they viewed the system’s positive and supportive tone as appropriate for the participant group. Other feedback such as number of questions per turn, and probing constraints were implemented in the prompt instructions.”

As the affiliative tone was not the primarily justification for the model choice, and as we did not upfront intend or anticipate interventional effects, we feel this emergent finding is best placed in the results and discussion. However, we defer to the editor as to whether this should be presented in the methods.

---

10. Data storage in Excel spreadsheets raises questions about data security, version control, and auditability, particularly for longitudinal qualitative data. Briefly justify this choice or clarify safeguards beyond password protection.

>>Author response

The technology system was approved under a University of Bristol Data Protection Impact assessment, which is a 10-page document covering data security aspects. We now include this as a supplementary file, referenced in the text. We hope that this will be an additional useful resource for researchers looking to develop and deploy similar systems. We now also include further details in the text, as follows:

> "The study received University ethics approval (ref: 14542-17493) and the technology system was approved under a University of Bristol Data Protection Impact assessment, to address issues of safety and data security. The full DPIA is provided in supplementary file (x). Steps to manage participant safety included choice of model, informing participants of risks in information sheet x, and a process of manual daily monitoring of conversational logs with a protocol to stop deployment and contact participants if any risks inappropriate exchanges was were noted. Data security processes included storing the chatlogs in a private encrypted database, being extracted into a password protected Excel file within a secure University digital environment, accessible only to the research team. Data in this file was pseudonymised. Participants were informed that data would be held for up to 10 years, and they have the right to request the deletion of their raw data up until the point of analysis. Other ethical considerations included giving particular attention to the accessibility of the participant information sheet and consent forms, given the potential for lower literacy levels in the participant group."

---

11. The scoring of relevance, clarity, and reflective depth is innovative, but the manuscript should be clearer about:

- **Inter-rater reliability beyond "resolving discrepancies," and**
- **How subjective judgments were managed, especially for reflective depth.**

>>Author response

We have added the following to provide greater transparency regarding the procedures within the coding approach that were implemented to support a consistent process - thereby reducing individual bias within an approach that does embrace some inherent subjectivity:

> "TK and SD each coded a single participant's chat-log data, meeting to discuss coding discrepancies and collaboratively form decision rules to guide further coding, per Schreier's consensus coding approach"

And

> "This work followed a pragmatic scoring approach, with scorers initially undertaking independent coding, before collaboratively discussing interpretative differences, prior to forming consensus in the form of scoring rules to guide scoring completion. This approach is consistent with consensus- based approaches in qualitative content analysis [38, 40]"

We also now include an additional supplementary file to provide additional transparency over the data in the chat logs and the ratings given by the research team – see S1 for scoring examples.

As we acknowledge there is an inherent degree of subjectivity in this coding process, the newly added positionality statement includes the following to increase transparency over the researchers involved in the coding process.

> "The research team comprised the first author, who was closely involved in the development, deployment, and evaluation of the CLEA system; a clinician who was more removed from system development, but had an interest in applied digital health research; and three senior researchers with expertise in digital health and qualitative methods. The analysis of interview data and the rating of chat logs involve an element of subjectivity, the team's diverse composition enabled multiple perspectives and ongoing sense-checking throughout these processes. This helped ensure that the analysis remained grounded in the data and was not unduly influenced by any single researcher's perspective.

We have also expanded the limitations section in the discussion to acknowledge the issues of subjectivity in the coding process:

> "... while procedures were implemented to improve the systematic and consistent rating of relevance, clarity, and utility (including independent double-coding and consensus development of decision rules), we acknowledge that some subjectivity is inherent in this form of evaluation and should be considered when interpreting the findings."

---

12. The reflective depth measure is described as "approximate," which is appropriate, but the authors should be careful not to overinterpret it later when discussing utility.

>>Author response

We currently state in the methods and discussion

"Our approximate heuristic of reflective depth averaged 0.46/1, indicating participants' typical single responses were sparing in cognitive or emotional depth."

> "… measures of response depth were necessarily approximate and used to provide some insight into the type of data elicited. While we used a specific, literature informed measure to approximate reflective depth, such an approach cannot fully characterise the concept of 'depth' in qualitative research or convey the value conversational data."

We have now amended wording where we refer to reflective depth, to try to ensure that claims are not over interpreted. This included amending wording to the following:

> "Replies in individual messages were often short and limited in depth, consistent with findings from related work [32]."

Results

The results are clearly structured and well supported by figures. However, several interpretive issues should be addressed.

13. The sample is described as underserved, but the demographic table suggests a relatively narrow population. Clarify whether deprivation refers to area-level context, program eligibility, or individual socioeconomic status.

>>Author response

We currently state "Three participants fell into the lowest decile of average gross household UK income, three in the second-lowest decile, two in the third, and one in the fourth.", which, we have change to "Three participants fell into the lowest decile of average gross household UK income, three in the second-lowest decile, two in the third lowest decile, and one in the fourth lowest decile." This clarifies that at the individual-level, all participants fell within the lower SES deciles, indicating that we did reach more disadvantaged participants at the individual-level. However, as eligibility was based on programme access, rather than individual level SES criteria, we agree that it is helpful to clarify this and have amended the wording throughout to reflect this. For example, changing the wording of "underserved population" to "community-based programme in an area of deprivation."

---

14. An 80% completion rate is reported as evidence of feasibility. However:

- **Deferred responses occurred in 33% of sessions, and**
- **Participants self-selected response timing.**
- **These findings are important and should be framed as conditional feasibility, not unqualified success.**

>>Author response

We have clarified in our reporting of feasibility results by adding:

> "Responses were classified as complete if participants replied to a prompt at any time; no specific timeframe was required for a response to be considered complete, and delayed responses were therefore considered complete. Accordingly, when we report feasibility, this is conditional-feasibility, reflecting participants' self-determined, organic use of the technology."

We also now reflect this in the discussion, stating

> "CLEA showed conditional longitudinal feasibility in this setting: completion rates (based on self-determined response times) were relatively high (80%), which compares favourably to other ILR methods…"

---

15. Themes around support, rapport, and non-judgement are compelling. However, these same qualities:

- **Risk conflating measurement with intervention, and**
- **Raise concerns about social desirability shaped by AI tone, not merely reduced by lack of a human.**
- **This tension is acknowledged later, but could be more clearly flagged in the results themselves.**

>>Author response

We agree that the distinction between CLEA being an observational research measurement tool versus an intervention is a central finding of this study. Aligning with health sciences conventions, we have reserved the Results section for reporting empirical findings (i.e., the qualitative data on interventional effects), using the Discussion to interpret these findings within the context of the wider literature. Accordingly, we would prefer to retain the more detailed, interpretative consideration of interventional effects in the Discussion. In the original version we included a dedicated section on reactivity and interventional effects, and we have now expanded on this, including further discussion of social desirability as a factor of AI tone and referring to this in the guidance for technological reflexivity:

> "Participants also reported effects consistent with support, reflection, and self-monitoring, suggesting that CLEA may in some cases have had reactive and even intervention-like effects on the phenomenon under study. While well reported in the intensive longitudinal methods literature (REF), this to our knowledge is the first recording of such effects occurring in AI-mediated qualitative research."

> "Our findings suggest that design choices in AI-mediated data collection systems such as CLEA should be treated as part of the method rather than merely implementation details. … design choices may extend beyond data production to shape the phenomenon under study itself through reactive or intervention-like effects. In this respect, AI model choice, user interface, and interactional guidance implemented through prompt engineering warrant particular reflexive attention in CLEA design. "

> "… such positivity can introduce non-neutral feedback, potentially inciting inauthentic positivity, reshaping self-perception over time, or producing interventional effects through relational support."

And in the conclusion, we now state:

> "Therein, decisions must consider epistemic implications, in how CLEA shapes accounts that are co-produced between participant and system, and how CLEA may, in some contexts, generate reactive or intervention-like effects on the phenomenon under study itself. We therefore urge explicit reflexivity in the development and reporting of future CLEA implementations, with careful attention to the technological configuration through which data are elicited."

We defer to the editor as to whether these interpretative points should also be briefly included in the Results section.

---

16. The finding that individual responses were often brief and shallow, while sessions aggregated meaningfully, is one of the manuscript's most important contributions. Emphasize this distinction more explicitly and avoid language that suggests CLEA reliably produces deep qualitative data at the individual response level.

>>Author response

In the results, we report findings at the level of individual messages to ensure transparency about the brevity of responses and limited depth at this level, we state:

> "Our approximate heuristic of reflective depth averaged 0.46/1, indicating participants' typical single responses were sparing in cognitive or emotional depth. Additionally, content was found to typically contain short and specific descriptions of recent behaviours, emotions and events."

And

> "The heatmap in Fig 6 shows that the presence of new codes was consistent over the 12-weeks, indicating the chat-agent was able to yield new and relevant insights per participant over the study period. This provides evidence that CLEA can gather useful insights into phenomena of interest as they fluctuate over time."

We agree that it is a key point to distinguish between limited depth at the single-message level versus greater insights aggregated over conversational sessions and over time. We have worked to clarify this this throughout the paper. In the abstract we now state:

> "whilst the responses in single messages were often brief, useful and relevant insights developed over the course of conversational sessions."

In the discussion we now state:

> "Replies in individual messages were often short and limited in cognitive or emotional depth, consistent with findings from related work [32]. However, our findings show that at the session-level, exchanges frequently yielded relevant and analytically useful material across the study period."

<u>Discussion</u>

The discussion is thoughtful and reflexive, but it occasionally moves beyond what the data can support.

17. Interventional effects. The manuscript states: "CLEA may have augmented the health program itself…" This is a plausible hypothesis, but it should be framed more clearly as speculative, not inferred.

>>Author response

We have amended the wording to differentiate findings from the data, and points of speculation. We now write:

> "Participants noted that interacting with CLEA helped consolidate learnings from programme sessions. Therein, the longitudinal compound effect of reactivity may have augmented the effect of the health programme itself over time."

---

18. Comparison with EMA, diaries, and interviews. Comparative positioning is useful, but readers should be reminded that:

- **No direct comparison study was conducted, and**
- **Claims are based on theoretical alignment and observed patterns, not head-to-head evidence.**

>>Author response

We have added the following at the start of our paragraph comparing CLEA to other ILRs:

> "CLEA was not directly compared with other methods in this study; therefore, no empirical evidence of its comparative position is available. Our preliminary findings, however, suggest that it is methodologically distinct from EMA, diary, and interview approaches. "

In the conclusion we state:

> "Compared with typical EMA designs, CLEA may enable greater interpretative richness through conversational interaction, but with reduced momentariness. Diary methods likely offer more open-ended, self-led reflection, but often at the cost of reduced topical relevance and increased analytic burden."

And we have now added the following caveat

> "However, comparative studies are needed to substantiate these comparisons."

---

19. Table 2 is a strong contribution. However, it would benefit from:

> **A brief explanation of how future researchers might operationalize reflexivity, not just consider it conceptually.**

>>Author response

Based on this comment and comments from other reviewers, we have amended table 4 and the accompanying text to include a more expanded consideration of reflexivity both conceptually and in terms of how it can operationalised. We have reworked the 'implications for design' to explicitly inform reflexive guidance and improve the links with Table 4.

> 'In light of these findings and surrounding literature, reflexivity in designing systems for AI-mediated qualitative research must extend beyond the human researcher to the technological configuration through which data are elicited. While qualitative data have

always been shaped by the tone, framing, and interactional style of human researchers, AI-mediated systems introduce a related but distinct form of influence, one embedded in model tendencies, interface, prompt scope, and conversational constraints. In this sense, AI-mediated bias should be understood not as separate from human bias, but as redistributing it within a technological layer. To address this, we build on the concept of technological reflexivity, introduced by Paulus et al. (2024) and applied to AI-assisted qualitative analysis by Prahl et al. (2025), and operationalise its relevance for AI-mediated data collection. Drawing on our findings, we highlight the key design domains, methodological considerations, and guidance for navigating technological reflexivity in future adaptations of CLEA (Table 4). We emphasise that each design choice involves trade-offs requiring explicit consideration.'

This includes changing table 4 columns of:

"design parameter", "methodological considerations and trade-offs for qualitative intensive longitudinal research" and "future work"

to

"design parameter", "questions for researchers", "Epistemic, reactive and interventional considerations" and "other methodological guidance"

Limitations

20. The limitations section is solid and honest, but two additional points should be emphasized:

- **The homogeneity of the sample limits claims about underserved populations broadly.**
- **The entanglement between CLEA and the program context makes it difficult to isolate CLEA's independent effects on engagement and disclosure.**

>>Author response

We have updated the limitations section of the discussion to address both these points, which now reads as follows:

"Several limitations should be considered when interpreting these findings. First, we sampled a relatively small and demographically homogeneous group, who were in stable housing and English speaking. Future work is required to understand the generalisability of the findings and how the technology would need to be adapted for other, more diverse populations, such as those in the most disadvantaged social situations, or non-English speakers."

and

"Finally, CLEA was embedded within an ongoing health programme, meaning that engagement with the system was possibly influenced by the programme context rather than by CLEA alone. The programme may have increased the perceived relevance of prompts and participants' motivation to engage, while CLEA may also have

reinforced programme learning through repeated reflection. This entanglement limits the extent to which observed engagement can be attributed to the system itself, and the extent to which interventional outcomes are a result of the intervention independent of CLEA. Future work should therefore investigate CLEA both within and outside programme settings to better understand how context influences engagement, data elicitation and the content of data itself. "

Conclusion

21. The conclusion appropriately calls for caution, but it still risks overselling CLEA as a broadly viable method. Reinforce that CLEA is best understood as:

- **A promising, configurable methodological approach,**
- **Most appropriate for specific research contexts and epistemological orientations,**
- **Complementary rather than substitutive.**

>>Author response

We have changed

> "These findings introduce CLEA as a viable, ecological method for longitudinal qualitative health research using conversational AI. CLEA was acceptable and engaging for a lower socioeconomic group involved in a weightloss programme. It was capable of capturing useful insights into their programme experience and associated behaviour change, over time."

to

> "Empirically, this particular configuration of CLEA was a feasible and acceptable qualitative intensive longitudinal research method deployed within a community-based programme, in an area of deprivation. While single messages were brief, across multiple messages within data collection sessions, it generated analytically useful insights into participants' programme experiences and associated behaviour change over time. Conceptually, we position CLEA as a configurable AI-mediated method in which design choices shape interaction with the system, data production, and, potentially, the phenomenon under study itself."

Also, the existing manuscript makes clear that the method is complementary to other methods, rather than substitutive:

> "Rather than replacing existing approaches, CLEA may best be understood as complementary to them. Hybrid EMA–CLEA designs may enable interpretive reflection anchored in momentary data, while CLEA-derived longitudinal insights could support recall and sense-making in retrospective interviews."

23rd April 2026

REVIEWER 2

This study offers an important conceptual contribution and demonstrates the potential of AI-mediated longitudinal qualitative research. However, in its current form, the epistemic positioning requires clarification.

1. While CLEA is acknowledged to not only collect data but also transform the studied process through reactivity and compound intervention effects, this needs to be more explicitly theorized. The distinction between programme effects and CLEA's reflexive intervention should be clearly articulated; as it stands, the boundary between "measurement" and "accompanying intervention" remains ambiguous.

>>Author response

We have provided more detail and consideration regarding possible intervention effects in the discussion:

> "Relatedly, participants' descriptions of engaging with CLEA suggest that accounts were shaped through these interactions. Conceptually, this supports treating CLEA-generated material as co-produced rather than as a neutral capture of pre-existing experience. Participants also reported effects consistent with support, reflection, and self-monitoring, suggesting that CLEA may in some cases have had reactive and even intervention-like effects on the phenomenon under study. While well reported in the intensive longitudinal methods literature (REF), this to our knowledge is the first recording of such effects occurring in AI-mediated qualitative research."

We have also added a column on "Epistemic, reactive and interventional considerations" in Table 4.

---

2. Although the co-constructed nature of the data is implied, its ontological and methodological implications require systematic treatment. In particular, the role of technological design as an epistemic actor should be placed within a more explicit theoretical framework.

>>Author response

We now provide explicit and comprehensive guidance (as per reviewer 1) for technological reflexivity in considering how design decisions in CLEA may affect data production, please see our additions in our reworked discussion:

> "In light of these findings and surrounding literature, reflexivity in designing systems for AI-mediated qualitative research must extend beyond the human researcher to the technological configuration through which data are elicited. While qualitative data have always been shaped by the tone, framing, and interactional style of human researchers, AI-mediated systems introduce a related but distinct form of influence, one embedded in model tendencies, interface, prompt scope, and conversational constraints. In this sense, AI-mediated bias should be understood not as separate from human bias, but as redistributing it within a technological layer. To address this, we build on the concept of technological reflexivity, introduced by Paulus et al. (2024) and applied to AI-assisted qualitative analysis by Prahl et al. (2025), and operationalise its relevance for AI-mediated data collection. Drawing on our findings,

> we highlight the key design domains, methodological considerations, and guidance for navigating technological reflexivity in future adaptations of CLEA (Table 4). We emphasise that each design choice involves trade-offs requiring explicit consideration."

---

3. The influence of model choice, prompt structure, and interface design on the nature of the data has not been empirically tested. This introduces the risk that findings are specific to the particular configuration used. Without systematic variation of these design parameters—or at minimum a stronger analytical justification—the generalizability and internal consistency of results appear limited. The study needs to make its conceptual and methodological framing more transparent, theoretically grounded, and robust. Such reinforcement would elevate the work into a reference-worthy contribution for the field.

>>Author response

This exploratory work highlighted the significance of design choices in data production. We explicitly acknowledge (see update to Limitations) that model choice, prompt structure, and interface design were not systematically tested but rather an output of co-design with charity partners. We present such systematic testing as an important avenue for future work:

> "Second, we operationalised only a single configuration of CLEA. Work is needed to examine the effects of reflexive adjustment of key design parameters, including model selection, prompt engineering, and user interface choice (see Table 2). Accordingly, our findings should be interpreted as evidence about one exploratory CLEA configuration deployed in a distinct health programme context, rather general validation of the CLEA method."

---

4. In addition, the discussion should consider AI-mediated guidance in relation to human researcher influence. In qualitative research, data is inherently co-produced between participant and researcher; the interviewer's tone, framing, and interaction shape responses. AI introduces a parallel, though distinct, type of influence: more systematic, scalable, and embedded in design parameters. The study should explicitly theorize similarities and differences between "technological bias" and "human bias" to clarify the epistemic stakes. Otherwise, AI's effect may be over- or under-problematized, and the human role implicitly assumed neutral, leaving methodological positioning incomplete.

>>Author response

As per point 2, and in-line with addressing reviewer 1's feedback, we have provided explicit and comprehensive guidance for technological reflexivity in considering how design decisions in CLEA may affect data production. Therefore, we address the point of considering guidance for how the researcher exerts influence over AI-mediated data collection. Theoretical differences between AI-mediated vs human-mediated qualitative data collection are interesting, and we have introduced this point in the discussion:

> "In light of these findings and surrounding literature, reflexivity in designing systems for AI-mediated qualitative research must extend beyond the human researcher to the technological configuration through which data are elicited. While qualitative data have always been shaped by the tone, framing, and interactional style of human researchers, AI-mediated systems introduce a related but distinct form of influence, one embedded in model tendencies, interface, prompt scope, and conversational constraints. In this sense, AI-mediated bias should be understood not as separate from human bias, but as redistributing it within a technological layer"

However, we believe development of a comprehensive theoretical framework that discusses in detail CLEA's influence on data and similarities or differences between human or technological bias are best addressed in future work that explores these factors explicitly. We are limited in the extent to which we can further theorise in this paper; reviewer 4 notes that 'the discussion is currently quite long'. Our Discussion focuses on presenting findings related to the study's key aims- namely the feasibility, acceptability, and utility of CLEA - situated within the context of the wider literature. Throughout the discussion, we have chosen to prioritise elaborating on points directly aligned with our aims, as well as providing a practical, reflexive tool for researchers considering AI-mediated qualitative data collection - rather than offering an extensive theoretical account of CLEA. We defer to the editor on whether a more expansive theoretical discussion should be included.

REVIEWER 3

Thank you for the opportunity to review the paper entitled "Conversational, Longitudinal, Ecological Assessment (CLEA): Exploring a new AI driven method for qualitative data collection in a behavioural health context".
The manuscript presents a genuinely innovative study and makes a valuable methodological contribution to the field of intensive longitudinal research. However, I believe several concerns regarding methodological transparency, analytical rigor, and reflexivity require attention before the manuscript can be considered for acceptance.

Abstract:
1. The abstract is clear and well-written.

Introduction:
2. The introduction is well-written and informative, providing a solid overview of the relevant literature.

3. Lines 168–173 offer a brief gesture toward reflexivity and the assumptions shaping

both the study and the tool development. This is a promising start, but it should not remain buried at the end of the Introduction. I encourage authors to develop this into a dedicated subsection within the Methods — addressing the composition of the research team, their theoretical assumptions, and how these shaped tool development, data collection, and analysis.

>>Author response

We have now included a dedicated positionality statement in the methods section, providing details about the composition of the team and theoretical assumptions:

> "The research team comprised the first author, who was closely involved in the development, deployment, and evaluation of the CLEA system; a clinician who was more removed from system development, but had an interest in applied digital health research; and three senior researchers with expertise in digital health and qualitative methods. The analysis of interview data and the rating of chat logs involve an element of subjectivity, the team's diverse composition enabled multiple perspectives and ongoing sense-checking throughout these processes. This helped ensure that the analysis remained grounded in the data and was not unduly influenced by any single researcher's perspective.
>
> Regarding the team's epistemological stance on AI-mediated qualitative research, we adopted a pragmatic stance that embraced the constructive nature of human and AI-mediated qualitative data generation, and the interpretive elements of qualitative analysis. In particular, the team recognised the need for reflexivity in CLEA's role in data- production, allowing for consideration of how researcher design choices might influence data produced by the method."

Methods:

4. The timeline of data collection remains unclear across Sections 2.2 and 2.3. While it is stated that participants could begin engaging with the tool from Week 3, the sampling frequency is never specified: Were participants prompted once per day? Every two days? How did answer deferral work in practice, and were reminders sent? These details are essential for evaluating the dataset and must be clarified.
5. It is unclear who initiated each conversational exchange. Did participants reach out to the CA, or did the CA send the first message? This has implications for understanding participant engagement and should be stated explicitly.

>>Author response

We have added the following to clarify the protocol, addressing points 4 and 5 above:

> "From enrolment until the end of the programme, participants received two CLEA prompts per week. Each prompt asked whether they had time to answer a short set of questions and included a 'Start Q&A' option to initiate the conversational flow. Participants could begin the session at any time until the next scheduled prompt

was sent. No reminders were provided; however, prompts remained available within WhatsApp and could be responded to at any time. Figure 2a–c shows the three prompt types used by the chat-agent: (2a) a post- session reflection prompt, typically sent on Tuesday after the Monday programme session, exploring participants' feelings about the session; (2b) a continuing post-session reflection prompt, exploring further aspects of the session experience; and (2c) a follow-up weekend prompt, exploring barriers and facilitators to behaviour change in the days following a programme session."

---

6. Pilot work with partners is mentioned but not adequately described. The authors should clarify the purpose and scope of the pilot, what was learned from it, and how this shaped the final design of the tool.

>>Author response

We have added the following to our Methods to address point 6, expanding on the pilot work with partners:

> "Technology choices were guided by principles of performance and safety, with consultation from charity partners. Considering performance, model selection was constrained by the technical landscape at the time of development (late 2023). Within this context, GPT-4 Turbo demonstrated reliability in consistently attending to information across longer prompts, follow system instructions reliably, maintain conversational coherence, and remain aligned to the intended research scope (Gavin, kuratov). GPT-4-Turbo was prioritised for prototype testing with charity partners in these respects, considering its preferable performance in terms of fidelity, which has implications for safety – for example, fidelity to adhering to guardrails like do not give medical advice. A prototype was set up in a test environment and connected to WhatsApp installed on university-owned mobile phones. Charity staff attended an in-person testing session, during which they interacted with the prototype live and provided feedback on its perceived suitability. In particular, they viewed the system's positive and supportive tone as appropriate for the participant group. Other feedback such as number of questions per turn, and probing constraints were implemented in the prompt instructions."
>
> "WhatsApp was identified as a suitable interface because partners already used WhatsApp Groups to communicate with participants during programme delivery, suggesting that it would be familiar and accessible within this context."

---

7. The figures displaying prompt samples are difficult to read. Beyond legibility, the authors should provide stronger rationale for the prompts used: Was prompt engineering applied? Were multiple prompt iterations tested? What criteria guided the final selection? This is an area of genuine methodological interest that deserves fuller treatment.

>>Author response

We have now reformatted the figures to make the text clearer.

Regarding prompt engineering, we state:

> "Our application contained specific prompt-based instructions to guide the AI in managing conversation flows with participants. Feedback from our charity partners was implemented within these prompt instructions following testing. Figures 2a-c detail the core operating instructions and research targets for the question-answer sessions. The chat-agent was instructed to act as a reflective interviewer, positioning it to elicit personal reflections. The prompt instructions prioritised understanding the participants' feelings, experiences, and behaviours. This, in effect, guided the content and frames of dialogue. Based on partner advice to manage participant burden and prioritise accessibility, the chat-agent was instructed to use simple language, ask no more than five questions and switch sub-topic after a maximum of three questions within a session. Additionally, the system was instructed to ask one question at a time and not lead participants. Finally, chat-agent-generated messages were capped at a maximum length of approximately 75 words. Throughout development, we sought to balance rich data collection with the burden placed on participants. Other choices and trade-offs might be considered more appropriate in different contexts of CLEA usage."

We have now also provide further details about the prompt engineering, to give it fuller treatment:

> "From enrolment until the end of the programme, participants received two CLEA prompts per week. Each prompt asked whether they had time to answer a short set of questions and included a 'Start Q&A' option to initiate the conversational flow. Participants could begin the session at any time until the next scheduled prompt was sent. Figure 2a–c shows the three prompt types used by the chat-agent: (2a) a post-session reflection prompt, typically sent on Tuesday after the Monday programme session, exploring participants' feelings about the session; (2b) a continuing post-session reflection prompt, exploring further aspects of the session experience; and (2c) a follow-up weekend prompt, exploring barriers and facilitators to behaviour change in the days following a programme session."

---

8. The authors may wish to include a sample of questions asked by the CA as a supplementary appendix. This would allow readers to evaluate the depth and style of the conversational data.

>>Author response

We now include sample text in results, and in supplementary materials.

---

9. The analysis section becomes confusing as it moves back and forth between the two data types (chat logs and interviews) and the different study objectives. I encourage authors to reorganize this section using sub-headings anchored to each data type or

analytical phase.

>>Author response

As Reviewer 1 states “the methods section is detailed and transparent” we believe that the complete reorganisation of sections is a matter of preference. However, we have actioned several changes that could make the analysis clearer. Firstly, we have added a short intro paragraph to the analysis section, to explicitly map the data analysis procedure to research aims and objectives. Secondly, we have seperated analysis elements and added sub-headings to help the reader navigate this section. We defer to the editor as to whether they feel this section needs to be more substantially reorganised.

---

10. Inter-rater reliability statistics (e.g., Cohen's Kappa or a comparable measure) are not reported. Additionally, the rationale for dividing coding responsibility, with one author scoring chat messages and another scoring participant responses, is unclear. Could this division have introduced systematic bias into the coding procedures? Please address this directly.

>>Author response

Please see our response to Reviewer 1, Comment 11, where we provide additional detail regarding the coding procedures, with reference to the consensus coding approach utilised [38, 40]. We also acknowledge the inherent subjectivity of the process and address this by including example text with ratings in table 1/ in a sup file to enhance transparency, a positionality statement outlining the researchers involved in the coding process, and a discussion of this methodological limitation in the manuscript. Finally regarding coding responsibilities, we state that at least two members of the research team score/code one full transcript of chat-agent messages or participant responses before meeting to compare, resolve discrepancies, with one member completing subsequent transcripts. This process of initial double coding, meeting to compare and reach consensus, with single coding using shared decision rules is a practical strategy to mitigate unchecked systematic bias.

Results:

11. All raters were members of the research team. The authors should reflect on how this may have influenced ratings of relevance, clarity, and utility, particularly given that participant responses were frequently brief. A brief acknowledgment of this limitation and its potential implications for data interpretation is warranted.

>>Author response

In response to comment 3, we now include a positionality statement. We believe this also helps address this comment, providing details about the members of the research team, their background, the diversity in perspectives, and how the multiple perspectives may have impacted rating:

> "The research team comprised the first author, who was closely involved in the development, deployment, and evaluation of the CLEA system; a clinician who was more removed from system development, but had an interest in applied digital health research; and three senior researchers with expertise in digital health and qualitative methods. The analysis of interview data and the rating of chat logs involve an element of subjectivity, the team's diverse composition enabled multiple perspectives and ongoing sense-checking throughout these processes. This helped ensure that the analysis remained grounded in the data and was not unduly influenced by any single researcher's perspective."

Whilst steps were taken to try and ensure a systematic and consistent approach to rating, we agree that there is a level of subjectivity in the ratings of relevance, clarity and utility. In response to comment 11 by reviewer 1, we now address this issue of subjectivity and potential bias, and have added a limitation in the discussion:

> "...whilst procedures were implemented to improve the systematic and consistent rating of relevance, clarity, and utility (including independent double-rating and consensus development of decision rules), we acknowledge that some subjectivity is inherent in this form of evaluation and should be considered when interpreting the findings."

---

12. At several points the manuscript overstates the depth and richness of participant responses, even while acknowledging their brevity. The authors should review every instance in which the utility or insight of responses is characterized, and ensure the language is proportionate to what the data actually supports.

>>Author response

We agree with this point, and also the point made in comment 16 by reviewer 1, "The finding that individual responses were often brief and shallow, while sessions aggregated meaningfully, is one of the manuscript's most important contributions". We have therefore worked to amend this throughout the paper - ensuring that depth and richness are not overstated and that the distinction is made between single message and conversational session richness.

---

13. The Results section, like the Methods, is dense and at times difficult to follow. The segmented presentation of themes contributes to this difficulty. Clearer organization would significantly improve readability.

>>Author response

Reviewer 1 comments that “The results are clearly structured and well supported by figures.”. Again, this may be a preference, and so we defer to the editor as to whether they feel the results should be restructured.

---

14. The team's reflexivity is largely absent from the Results. Given the researchers' centrality to both tool design and coding, the authors should more explicitly acknowledge how their positioning may have shaped the analysis and interpretation of findings.

>>Author response

In response to comment 3 – and in line with feedback from reviewer’s 1 and 2 - we now include a positionality statement in the methods section. This covers a discussion of the team’s composition and how this may have shaped data analysis.

---

15. Line 541: Please revise the phrase "to promote a sense of non-judgemental, enabling".

>>Author response

This has been changed to

“

“…to promote a sense of non-judgemental interaction, enabling…”

Discussion:

16. As in the Results, the Discussion at times overstates the usefulness and depth of participant answers. Given how little of the actual response content is presented, strong characterizations of participant insight are difficult to substantiate. The authors should moderate these claims accordingly.

>>Author response

As detailed in response to comment 12, we have worked to amend discussions of usefulness and depth, ensuring these are not overstated and that the distinction is made between single message and conversational session richness.

Further, in response to other comments, we now include example text in supplementary file 1, which enhances transparency over the response content and ratings.

---

17. The claim that "participants appeared to co-construct accounts with the chat-agent" (lines 582–583) is only partially supported by the findings presented. The authors should either provide stronger evidentiary grounding for this claim in the Results section, or revise the language in the Discussion to reflect the degree of support the data actually provides.

>>Author response

Regarding this claim, we have revised to the below, explicitly tying the claim to the data from which it is supported. Furthermore, as above, we have added conversational data in supplementary material to support the findings.

> "Relatedly, participants' descriptions of engaging with CLEA suggest that accounts were shaped through these interactions. Conceptually, this supports treating CLEA-generated material as co-produced rather than as a neutral capture of pre-existing experience."

Implications of Design Choices

18. The discussion of design margins (e.g., a maximum of five questions per session) reads as a methodological description rather than a discussion of implications. This material is better placed in the Methods section, where it would help clarify the data collection procedures. The Implications section should focus on what these choices mean for the interpretation of findings and for future researchers adopting similar approaches.

>>Author response

We have now significantly revised table 4 to provide a more robust discussion of implications and providing guidance for technological reflexivity in the future design or extension of an AI-mediated longitudinal qualitative tool. As per reviewer 1, we have operationalised our findings in this section to provide practical support to future researchers looking to utilise CLEA; we therefore believe the table is best placed in the discussion section, but defer to the Editor.

Positioning CLEA Relative to Other Intensive Longitudinal Methods

19. Line 691 contains a typo: "CLEA appers Like EMA, CLEA can be" — please revise.

>>Author response

Corrected

Overall, this is a very interesting and innovative study. Mostly, I suggest authors to further include strengthen reporting, focusing on acknowledging reflexivity/positionality; clarify the data collection timeline, sampling procedures, and initiation protocol; and reorganize the Methods and Results sections for clarity.

REVIEWER 4

Abstract

1. The abstract currently jumps straight into the design and findings of the study, but it would benefit from including some background literature and the gap in the literature.

>>Author response

Reviewer 1 notes "The abstract is generally clear and informative" and reviewer 3 notes: "The abstract is clear and well-written". While we believe that adding in literature into the abstract is unnecessary, we acknowledge a smoother funnel into the findings would be appropriate. We have added the following to the abstract:

"Qualitative research is key to gaining rich insights into health phenomena. Qualitative interviews allow in-depth, adaptive data collection but are resource-intensive and often infrequent and removed from real-time experience. Intensive longitudinal research (ILR) methods include frequent sampling, closer to events, but are static, varying from constrained (EMA) to unstructured (diaries). Integrating conversational AI into ILR may build on strengths from interviews and IRL, offering adaptive, frequent and in-the-moment data collection.

---

2. Highlighting the theoretical and practical implications of the study would also strengthen this section.

>>Author response

To indicate the practical contributions of the paper, we have amended:

> "... considering design implications for future iterations."

To

> "... providing guidance on design implications and reflexivity for AI-mediated qualitative research tools for health researchers looking to implement or extend the method."

---

3. Some parts of the author summary could be integrated into the abstract to provide a more coherent overview of what was done and to give context for the study.

We have amended the abstract per comment 1 and 2. We are hesitant about including more detail within the abstract, considering Reviewer 1 notes “The abstract is generally clear and informative” and reviewer 3 notes “The abstract is clear and well-written". We defer to the Editor as to whether elements of the author summary are necessary to be intergrated into the abstract.

---

4. Introduction

Certain sections (e.g., lines 72–74) are missing references.Lines 74–76 read awkwardly and may
a typo; lines 72–79 overall lack references and need revision for clarity.Lines 82–83 mention “retrospective,” but it isn’t defined.Lines 86–87, 103–105, 111–112, 114–115, and 160–164 are also missing references.
Careful attention to these references and smoothing out awkward sentences will make the introduction more coherent and credible.

>>Author response

Reviewers 1 and 3 consider the introduction to be strong and well written. Some of the notes from reviewer 4 are more stylistic, but we welcome the feedback in making the writing more readable and rigorously referenced. As such, we have made the following edits:

References added to:

Lines 72-74 (wording smoothened also) / Lines 72-79 / Lines 103-105

‘Retrospective interview’ - removed ‘retrospective’

Lines 111-112 is a general statement that is established in the digital health and qualitative research communities; there is therefore no need for a reference here.

Lines 160-164 are introducing CLEA as a research method, we do not believe there is a need for a reference here:
“We position CLEA as a high-level ILR longitudinal research method that, through specific design choices, can be adapted to conduct longitudinal research with varying qualities of EMA or diary method depending on requirements of the respective study context. In this work, we explore the use of CLEA for qualitative data collection in a behavioural health context.”

Methods

5. Objectives and aims should be clearly stated in the Introduction section.

>>Author response

In the introduction we provide an overview of aims, we state:

> “Our research aims in this work were to establish A) feasibility of the tool B) acceptability of the tool to users, and C) the utility of the data captured over this period.”

In the methods we provide a more detailed description of aims and objectives, we state:

> “Aim A: To determine the feasibility of CLEA for real-world longitudinal qualitative data collection, in terms of instrument fidelity and data completeness
>
> > Objective A1: To assess chat-agent question clarity and relevance to the i nstructed research target (fidelity)
> >
> > Objective A2: To assess participant longitudinal engagement with CLEA, in terms of number of data collection sessions responded to, sessions completed, frequency of deferred responses and response length (within and across participants).
>
> Aim B: To evaluate the acceptability and perceived usability of CLEA
>
> > Objective B1: Explore participant experiences, perceptions and attitudes to chat-based interactions with CLEA
>
> Aim C: To determine the quality and utility of longitudinal qualitative data gathered using CLEA
>
> > Objective C1: To understand CLEA’s ability to collect new and insightful data over time per participant
> >
> > Objective C2: To assess clarity, relevance, and approximate reflective depth in participant responses to CLEA’s questions
> >
> > Objective C3: To explore participant experiences and perceptions to the provision of meaningful data over time, and the alignment to observed behaviours in chat-logs"

We think this is a clear structure. But we defer to the editor as to whether they would prefer us to move the expanded aims and objectives to the introduction rather than the methods.

---

6. Including a detailed procedure section would enhance replicability and make it easier for readers to understand exactly how the study was conducted.

>>Author response

We have added a section “CLEA participant procedures” which details the protocol for participants interacting with CLEA. Furthermore, in line with feedback from reviewer 3, we have updated the analysis section support the reader in navigating the procedure, and clearly signposted how each sub-section answers specific research objectives. We feel this is a suitable amend, considering reviewer 1 describes the methods as ‘detailed and transparent’.

Results

7. Consider placing excerpts into a table under each theme. This will improve readability and make it easier to define and illustrate the themes.

>>Author response

We appreciate that there are different stylistic preferences for how to present quotes within thematic analysis. Reviewers 1 notes “The results are clearly structured”. Our preference is to keep the quotes where they are – integrated into paragraphs - but we defer to the editor as to whether these should be moved. However, we have added some examples of the data at the start of the results section, to help provide perspective to the reader as to the format and content of interactions.

Discussion

8. Linking the discussion of themes to past research will strengthen this section and highlight the significance of your findings.

>>Author response

Throughout our summary of findings, we have now connected our findings to broader literature. Please see updates in the respective section.

---

9. The discussion is currently quite long and the use of subheadings could be reconsidered. Condensing this section and focusing on how findings relate to broader literature, rather than methodological comparisons, will improve clarity and impact.

>>Author response

In response to this comment we have have removed several sub-headings to improve flow in the discussion. We have related our key findings to broader literature in the summary of findings. However, considering work explores CLEA as a technology expansion of ILR methods, it feels appropriate to use the discussion to reflect on methodological comparisons.

23rd April 2026

References

1. Creswell, J. W. (2007). Qualitative Inquiry and Research Design: Choosing Among Five Approaches. Sage Publications
2. Dawadi, S., Shrestha, S., & Giri, R. A. (2021). Mixed-Methods Research: A Discussion on its Types, Challenges, and Criticisms. Journal of Practical Studies in Education, 2(2), 25-36. DOI: https://doi.org/10.46809/jpse.v2i2.20
