# Supplementary material for: Conversational, Longitudinal, Ecological Assessment (CLEA): Exploring a new AI-driven method for qualitative data collection in a behavioural health context
Source: PLOS Digit Health. 2026 May 27;5(5):e0001216. doi: 10.1371/journal.pdig.0001216 (PMC13215495; doi:10.1371/journal.pdig.0001216)
Supplement: Attachment — Submitted filename: Reviewer Report.docx [file pdig.0001216.s004.docx]

**Reviewer Overview**

Thank you for the opportunity to review this manuscript, *“Conversational, Longitudinal, Ecological Assessment (CLEA): Exploring a new AI-driven method for qualitative data collection in a behavioural health context.”*

This manuscript addresses an important and timely methodological question within digital health, qualitative methods, and human–AI interaction: how conversational AI may function as a tool for longitudinal qualitative data collection in real-world health contexts. The authors propose CLEA as a novel method and present an exploratory deployment within a community-based weight management program. The work is thoughtful, well written, and grounded in relevant literature across EMA, diary methods, and conversational agents. At the same time, because the manuscript makes methodological claims rather than reporting a conventional intervention outcome, the standards for conceptual clarity, transparency, and evidentiary alignment are especially high. Several sections would benefit from sharper framing, clearer differentiation between feasibility, acceptability, and utility, and more cautious interpretation of findings given the small, homogeneous sample and the entanglement between data collection and intervention effects.

My comments below are intended to strengthen the manuscript’s clarity, rigor, and contribution, not to undermine its value.

**Major Comments to the Authors**

**Abstract**

The abstract is generally clear and informative, but it currently overstates the strength and generalizability of the findings given the exploratory nature of the study.

- The abstract states: “Together, these findings demonstrate initial feasibility and acceptability of CLEA for longitudinal qualitative data collection in an underserved population.”

While the population is described as underserved, the sample is small (N=9) and predominantly White British (n=7). The authors should either:

- Clarify what is meant by “underserved” in this specific context, or
- Use more cautious language (e.g., “participants enrolled in a community-based program in an area of deprivation”).
- The abstract also claims CLEA can “elicit meaningful, contextually grounded insights consistently over time.”
  Given that reflective depth scores averaged 0.46/1, this claim would benefit from qualification, for example, by distinguishing session-level aggregation from individual response depth, which is discussed later in the paper but not reflected in the abstract. Tighten claims to more closely reflect the exploratory design and analytic results.

**Introduction**

The introduction is strong in its methodological grounding and provides a solid overview of existing longitudinal qualitative methods. However, several improvements would strengthen its contribution.

1. Positioning of CLEA as a “new method” The manuscript introduces CLEA as a “novel” method, but novelty is sometimes implied rather than explicitly demonstrated. The authors should more clearly articulate:
   - What CLEA enables that existing EMA + diary hybrids do not, and
   - Whether the novelty lies primarily in the technology (LLM-based CA), the interactional structure, or the methodological framing.
2. The introduction states: “There has been limited investigation into the deployment of CAs for longitudinal, in-the-wild qualitative data collection…” This is accurate, but the authors could strengthen the argument by:
   - Explicitly stating what remains unknown (e.g., engagement decay, data quality trade-offs, reactivity), and
   - Mapping each unknown directly to the stated aims (feasibility, acceptability, utility).
3. The manuscript references a “constructivist consideration of knowledge production” later, but this stance is not clearly introduced here. Because epistemology directly shapes the interpretation of AI-mediated data collection, it would be helpful to:
   - Briefly signal the authors’ epistemological positioning earlier, and
   - Explain how this stance justifies CLEA’s conversational and potentially interventional qualities.

**Methods**

Overall, the methods section is detailed and transparent, which is a major strength. However, several areas require clarification or strengthening.

4. The manuscript describes the study as “mixed-methods,” but the integration between quantitative engagement metrics and qualitative interview data is not fully articulated. Clarify whether this is:
   - A convergent mixed-methods design,
   - A primarily qualitative study with descriptive quantitative support, or
   - An exploratory methodological evaluation.
5. Eligibility criteria include “stable housing” and English-speaking participants. These criteria may systematically exclude individuals most affected by digital exclusion or instability. Acknowledge how these criteria shape the conclusions about accessibility and acceptability.
6. Participants joined at different weeks (weeks 3–5). While acknowledged later as a limitation, this staggered entry has implications for longitudinal comparability that could be more clearly addressed upfront.
7. CLEA System and AI Model Choice This is one of the strongest sections of the paper, but also one of the most methodologically consequential. The authors state:

“The GPT4-Turbo model tends towards a positive, affiliative tone… This tendency… was desirable…”

This choice is defensible, but it is also interventional, as later acknowledged. The methods section would benefit from:

- A clearer justification of why an affiliative model was preferable to a more neutral one for a *data collection* study, and
- An explicit acknowledgement that this choice may shape participant affect and responses from the outset.

8. Data storage in Excel spreadsheets raises questions about data security, version control, and auditability, particularly for longitudinal qualitative data. Briefly justify this choice or clarify safeguards beyond password protection.
9. The scoring of relevance, clarity, and reflective depth is innovative, but the manuscript should be clearer about:
   - Inter-rater reliability beyond “resolving discrepancies,” and
   - How subjective judgments were managed, especially for reflective depth.
10. The reflective depth measure is described as “approximate,” which is appropriate, but the authors should be careful not to overinterpret it later when discussing utility.

**Results**

The results are clearly structured and well supported by figures. However, several interpretive issues should be addressed.

11. The sample is described as underserved, but the demographic table suggests a relatively narrow population. Clarify whether deprivation refers to area-level context, program eligibility, or individual socioeconomic status.
12. An 80% completion rate is reported as evidence of feasibility. However:
    - Deferred responses occurred in 33% of sessions, and
    - Participants self-selected response timing.

These findings are important and should be framed as conditional feasibility, not unqualified success.

13. Themes around support, rapport, and non-judgement are compelling. However, these same qualities:
    - Risk conflating measurement with intervention, and
    - Raise concerns about social desirability shaped by AI tone, not merely reduced by lack of a human.

This tension is acknowledged later, but could be more clearly flagged in the results themselves.

14. The finding that individual responses were often brief and shallow, while sessions aggregated meaningfully, is one of the manuscript’s most important contributions. Emphasize this distinction more explicitly and avoid language that suggests CLEA reliably produces deep qualitative data at the individual response level.

**Discussion**

The discussion is thoughtful and reflexive, but it occasionally moves beyond what the data can support.

15. Interventional effects. The manuscript states: “CLEA may have augmented the health program itself…” This is a plausible hypothesis, but it should be framed more clearly as speculative, not inferred.
16. Comparison with EMA, diaries, and interviews. Comparative positioning is useful, but readers should be reminded that:
    - No direct comparison study was conducted, and
    - Claims are based on theoretical alignment and observed patterns, not head-to-head evidence.
17. Table 2 is a strong contribution. However, it would benefit from:
    - A brief explanation of how future researchers might *operationalize* reflexivity, not just consider it conceptually.

**Limitations**

The limitations section is solid and honest, but two additional points should be emphasized:

- The homogeneity of the sample limits claims about underserved populations broadly.
- The entanglement between CLEA and the program context makes it difficult to isolate CLEA’s independent effects on engagement and disclosure.

**Conclusion**

The conclusion appropriately calls for caution, but it still risks overselling CLEA as a broadly viable method. Reinforce that CLEA is best understood as:

- A promising, configurable methodological approach,
- Most appropriate for specific research contexts and epistemological orientations,
- Complementary rather than substitutive.
