# Supplementary material for: Conversational, Longitudinal, Ecological Assessment (CLEA): Exploring a new AI-driven method for qualitative data collection in a behavioural health context
Source: PLOS Digit Health. 2026 May 27;5(5):e0001216. doi: 10.1371/journal.pdig.0001216 (PMC13215495; doi:10.1371/journal.pdig.0001216)
Supplement: Attachment — Submitted filename: PDIG-D-26-00092.docx [file pdig.0001216.s005.docx]

Manuscript ID: PDIG-D-26-00092

Thank you for the opportunity to review the paper entitled “Conversational, Longitudinal, Ecological Assessment (CLEA): Exploring a new AIdriven method for qualitative data collection in a behavioural health context”.

The manuscript presents a genuinely innovative study and makes a valuable methodological contribution to the field of intensive longitudinal research. However, I believe several concerns regarding methodological transparency, analytical rigor, and reflexivity require attention before the manuscript can be considered for acceptance.

--

**Abstract:**

1. The abstract is clear and well-written.

**Introduction:**

2. The introduction is well-written and informative, providing a solid overview of the relevant literature.

3. Lines 168–173 offer a brief gesture toward reflexivity and the assumptions shaping both the study and the tool development. This is a promising start, but it should not remain buried at the end of the Introduction. I encourage authors to develop this into a dedicated subsection within the Methods — addressing the composition of the research team, their theoretical assumptions, and how these shaped tool development, data collection, and analysis.

**Methods:**

4. The timeline of data collection remains unclear across Sections 2.2 and 2.3. While it is stated that participants could begin engaging with the tool from Week 3, the sampling frequency is never specified: Were participants prompted once per day? Every two days? How did answer deferral work in practice, and were reminders sent? These details are essential for evaluating the dataset and must be clarified.

5. It is unclear who initiated each conversational exchange. Did participants reach out to the CA, or did the CA send the first message? This has implications for understanding participant engagement and should be stated explicitly.

6. Pilot work with partners is mentioned but not adequately described. The authors should clarify the purpose and scope of the pilot, what was learned from it, and how this shaped the final design of the tool.

7. The figures displaying prompt samples are difficult to read. Beyond legibility, the authors should provide stronger rationale for the prompts used: Was prompt engineering applied? Were multiple prompt iterations tested? What criteria guided the final selection? This is an area of genuine methodological interest that deserves fuller treatment.

8. The authors may wish to include a sample of questions asked by the CA as a supplementary appendix. This would allow readers to evaluate the depth and style of the conversational data.

9. The analysis section becomes confusing as it moves back and forth between the two data types (chat logs and interviews) and the different study objectives. I encourage authors to reorganize this section using sub-headings anchored to each data type or analytical phase.

10. Inter-rater reliability statistics (e.g., Cohen's Kappa or a comparable measure) are not reported. Additionally, the rationale for dividing coding responsibility, with one author scoring chat messages and another scoring participant responses, is unclear. Could this division have introduced systematic bias into the coding procedures? Please address this directly.

**Results:**

11. All raters were members of the research team. The authors should reflect on how this may have influenced ratings of relevance, clarity, and utility, particularly given that participant responses were frequently brief. A brief acknowledgment of this limitation and its potential implications for data interpretation is warranted.

12. At several points the manuscript overstates the depth and richness of participant responses, even while acknowledging their brevity. The authors should review every instance in which the utility or insight of responses is characterized, and ensure the language is proportionate to what the data actually supports.

13. The Results section, like the Methods, is dense and at times difficult to follow. The segmented presentation of themes contributes to this difficulty. Clearer organization would significantly improve readability.

14. The team's reflexivity is largely absent from the Results. Given the researchers' centrality to both tool design and coding, the authors should more explicitly acknowledge how their positioning may have shaped the analysis and interpretation of findings.

15. Line 541: Please revise the phrase "to promote a sense of non-judgemental, enabling".

**Discussion:**

16. As in the Results, the Discussion at times overstates the usefulness and depth of participant answers. Given how little of the actual response content is presented, strong characterizations of participant insight are difficult to substantiate. The authors should moderate these claims accordingly.

17. The claim that "participants appeared to co-construct accounts with the chat-agent" (lines 582–583) is only partially supported by the findings presented. The authors should either provide stronger evidentiary grounding for this claim in the Results section, or revise the language in the Discussion to reflect the degree of support the data actually provides.

**Implications of Design Choices**

18. The discussion of design margins (e.g., a maximum of five questions per session) reads as a methodological description rather than a discussion of implications. This material is better placed in the Methods section, where it would help clarify the data collection procedures. The Implications section should focus on what these choices mean for the interpretation of findings and for future researchers adopting similar approaches.

**Positioning CLEA Relative to Other Intensive Longitudinal Methods**

19. Line 691 contains a typo: "CLEA appers Like EMA, CLEA can be" — please revise.

Overall, this is a very interesting and innovative study. Mostly, I suggest authors to further include strengthen reporting, focusing on acknowledging reflexivity/positionality; clarify the data collection timeline, sampling procedures, and initiation protocol; and reorganize the Methods and Results sections for clarity.
